# RD internationalization, domestic technology alliance, and innovation in emerging market

**DOI:** 10.1371/journal.pone.0252669

**Published:** 2021-06-25

**Authors:** Jingjing Li, Gang Liu, Zihan Ma

**Affiliations:** 1 School of Economics and Management, Wuhan University, Wuhan, Hubei, China; 2 School of Business Administration, Hubei University of Economics, Wuhan, Hubei, China; University of Almeria, SPAIN

## Abstract

Although R&D internationalization plays an important role in enterprises’ globalization, few studies explore the mechanism of R&D internationalization and emerging market companies’ innovation, or the relationship between R&D internationalization, domestic technology alliances and absorptive capacity. How does the R&D internationalization of emerging market enterprises affect the innovation of those enterprises? Under fierce market competition, do absorption capacity and domestic technology alliances have a significant impact on enterprise innovation? From the perspective of the knowledge-based view, this paper studies 185 enterprises undergoing R&D internationalization in China from 2012 to 2017, using high-dimensional Poisson fixed effects model, we use instrumental (HDFE IV) estimation to explain the impact of R&D internationalization on the innovation of the parent company and the mechanism behind it. The study finds that R&D internationalization positively promotes the parent company’s innovation, and domestic technology alliances and absorptive capacity play a partial mediator role in R&D internationalization. In the face of fierce market competition, domestic technical alliances play a significant role in promoting enterprise innovation, while absorptive capacity plays a negative role in promoting enterprise innovation with the moderating effect of market competition.

## 1. Introduction

The springboard theory and 3L theory illustrate the phenomenon of innovation catch-up of emerging market enterprises [1, 2]. Different from the internationalization activities carried out by developed countries based on the expansion of the advantages of their home countries, the motivation of the internationalization of enterprises in emerging markets is mostly based on the escape from the inferior institutional system and the pursuit of advanced technology. They quickly learn mature technologies, management skills and organizational patterns of developed countries through mergers and acquisition and the establishment of overseas R&D centers, as to gain a favorable competitive position in the fierce global competition [1–4]. As the most direct way for enterprises to acquire technology [5–7], R&D internationalization plays a crucial role in the innovation catch-up of emerging market enterprises. However, there is little research on the mechanism of R&D internationalization in the existing literature on the internationalization of emerging market firms [8, 9], after acquiring international knowledge, how can emerging market companies apply these skills and knowledge to their own use to achieve innovation catch-up? Ignoring this problem may lead to a one-sided and simple understanding of innovation catch-up in emerging market enterprises. Ignoring these problems in management may be detrimental to the optimal allocation of resources.

In terms of research related to R&D internationalization, some scholars have suggested that through R&D internationalization, multinational enterprises could take advantage of the parent companies’ new technology and management mode to expand the international market [10, 11], or through the international R&D subsidiaries to acquire the advanced knowledge assets of the host country, coordinate and integrate the global R&D network to achieve knowledge transfer and sharing, so as to improve the international competitive advantage of the enterprise [9, 12–14], especially for the multinational enterprises in emerging market, the R&D internationalization is an important "springboard" for them to catch up and even surpass the enterprises in developed countries [1]. To be specific, different motivations of R&D internationalization may bring different results: some scholars have found that the international technology acquisition on the performance of parent companies is more positive than that of international technology utilization [15, 16]; some scholars have studied the entry mode of R&D internationalization and come to the conclusion that different entry modes meant that enterprises’ internationalization required different human and material resources, took different risks and had different influences on the performance of enterprises [17, 18]; some other scholars have believed that the geographical dispersion of R&D internationalization would affect the international innovation process of enterprises and thus affect the performance of enterprises [17, 19–21]. When concerns to the relevant capabilities of R&D internationalization, Jian & Myles proposed that when an enterprise had a high absorption capacity, R&D internationalization had a significant promoting effect on the innovation of the parent company [22], meanwhile, absorption capacity is also important for external mergers and profit acquisition [23–25]. When studies refer to the domestic scene, some scholars have researched the relationship between the international R&D and the domestic R&D. Belderbos et al. used 156 enterprise’s panel data in the years 1995–2002 from Europe, the United States and Japan, and found that based on economies of scale, economies of scope, cost of cooperation and domestic embeddedness, compared with R&D internationalization, enterprises preferred domestic R&D [26]. Belderbos et al. also used dynamic panel analysis of 4,038 enterprises in the Netherlands, and concluded that compared with technology-leading enterprises, R&D internationalization of technology-laggard enterprises was more likely to increase the productivity of domestic R&D [27].

However, these studies mainly focus on developed countries, and lack of research on the relationship between R&D internationalization and innovation performance of muti-national enterprise (MNE) in emerging market. More importantly, how domestic factors like the domestic technology alliance, the absorptive capability of parent company, the fierce market competition of the home country affect the innovation of enterprise has not been emphasized in the international business literature, while the impact of innovation on enterprise performance is crucial [28], studying the internal mechanism of the impact of R&D internationalization on innovation of emerging market enterprise will not only help us understand the innovation path of emerging market firms, but also help us understand the key factors that contribute to their business performance.

For developed countries, the conclusion is still ambiguous on whether overseas research and development boosts enterprise innovation. The mainstream view holds that R&D internationalization promotes enterprise innovation and promotes innovation overflow between the parent company and its foreign subsidiaries [29, 30]. The promotion is crucial to maintaining the competitive advantage of enterprises in the international market [31]. However, some scholars have suggested that the geographical dispersion of R&D activities is negatively correlated with the innovation of enterprises [32]. Some scholars have indicated that the R&D internationalization is non-linear to the innovation of the parent company. For example, Hsu et al. believed that the investment of R&D internationalization subsidiaries in host countries was affected by their adaptation cost, coordination cost and supervision cost, and the ratio of cost to investment income showed a trend of first increasing and then decreasing. Reversely, there was a U-shaped relationship between R&D internationalization business and the innovation of the parent company [33]. Chen et al. pointed out that the autonomy of the R&D internationalization subsidiary was transformed with the periodical changes of the "centrifugal", "transformation" and "centralization", and the influence of R&D internationalization on the innovation of the parent company was an "S-type" curve [34]. Different from developed countries, emerging market enterprises mainly concentrate their overseas investment in developed countries, and the investment process is more radical and rapid. They regard R&D internationalization as a favorable mean to learn advanced technologies from developed countries [35], therefore, it is urgent for us to study the relationship between R&D internationalization and parent companies’ innovation as well as its internal mechanism of emerging market enterprises. However, from the perspective of the combination of international R&D and domestic factors, the existing researches mostly study international R&D and domestic R&D separately [10, 36, 37], ignoring the role of innovation factors within the home country, this may lead to the simplification and superficial of research innovation problems, considering the innovation environment of the home country, we combined the R&D internationalization with local factors such as the absorptive capacity of the parent company, domestic technology alliance and the intensity of local market competition, it is beneficial for us to explore the key role of local factors in the process of R&D internationalization, so as to have a more comprehensive understanding of the innovation process catch-up of emerging market enterprises.

While China is in a period of market reform [38], the domestic technology alliances and absorptive capacity are key factors of emerging market enterprises on their innovation. Studying the mechanism of RD internationalization and the domestic innovation factors is of great significance for us to understand the innovation leap of latecomers such as China, which is in an economic and social transformation period, and research on the impact of market conditions on firm R&D activities is very important in a country in economic transition such as China, but the existing literatures focus on institutional and cultural studies [39–41]. The effect of market competition on absorptive capacity and technology alliance of enterprises is ignored.

Through empirical research, we found that R&D internationalization not only directly promoted the innovation of enterprises, but also indirectly promoted the innovation of enterprises through their own absorption capacity and the help of domestic technology alliances, we revealed that for Chinese enterprises in the period of social and economic change, the fierce market competition was more advantageous to the domestic technology alliances aspect of enterprise innovation, and absorptive capacity in the fierce market competition environment negatively promoted enterprise innovation in the short term, which is very different from developed countries.

In recent years, as the world’s largest emerging economy and the leading destination of foreign direct investment [42], China is in an emerging economy with the most rapid economic and technological development, and plays an important role in the international strategies of many multi-national corporations [43–45], so we select Chinese listed companies as our empirical database, we collect the information of 185 companies during the year 2012 to 2017 as our dataset, we chose this sample as empirical dataset because these enterprises have fewer missing values in the selected years, and they also have more overseas R&D activities and domestic R&D activities, the selection of these enterprises can maintain the integrity and authenticity of our data and maximize the sample size. Specifically, this is the final sample that we had passed through layers of criteria and screening in the database. Listed companies have relatively complete information and accurate information disclosure, so we chose listed companies as our overall samples. Among these samples, we selected some industries with frequent overseas research and development activities as our basic database, because this can maximize our sample size. First, we excluded those companies that have been at a loss during all the years and have abnormal financial conditions. Second, we excluded companies that have set up subsidiaries for tax avoiding purposes. Thirdly, we excluded enterprises whose subsidiary business is unknown. Finally, we excluded samples with a large number of missing values and outliers. See more details in the “*Variables selection and measurement*” part. The theoretical and empirical contributions of this paper are as follows:

First, our research is a useful supplement and extension to the existing international investment theories. It not only confirms the motivation of technology-seeking foreign investment of emerging market enterprises, but also elaborates the internal mechanism of its effect on innovation, which is a gap in the existing literatures and helps us better understand the rise process of emerging market enterprises. Compared with the purpose of asset-exploiting international investment in developed countries [35, 46, 47], cross-border investment of emerging market enterprises is totally different, these enterprises hope to obtain core competitive resources through rapid internationalization [1, 48]. R&D internationalization is the most direct and rapid way to acquire technological resources. Existing literature focuses on the motivation and expansion methods of internationalization of emerging market firms [49, 50], lack of mechanism research on the internationalization of emerging market enterprise. Studying the mechanism of the R&D internationalization of emerging market enterprise is of great significance for us to understand the catch-up path of emerging market firms and to supplement and expand the existing international business theories, at the same time, the research on the mechanism of RD internationalization also helps managers to pay attention to the factors that multinational enterprises may ignore in the process of internationalization: the absorptive capacity of enterprise and local stakeholders, so as to better promote enterprise innovation and performance.

Second, our research has enriched the knowledge-based theory and absorptive capacity theory. The purpose of R&D internationalization is to acquire valuable knowledge and technology. However, such knowledge and technology are more difficult to transfer than domestic knowledge because they are deeply embedded in the social environment where they located in [51, 52]. The institution, the level of economic development and cultural customs of the host country are quite different from those of emerging market enterprises, so absorbing such knowledge not only requires the parent company to have higher ability to distinguish, integrate and digest knowledge [53], but also needs the assistance of local stakeholders. Understanding the mediating role of absorptive capacity and local technology alliance in R&D internationalization and innovation can help us better understand the absorption path of advanced technology and knowledge, thus, the theory of absorptive capacity is enriched. Meanwhile, the degree of local market competition and domestic technology alliance belongs to local environmental factors. According to the knowledge-based view, as a social entity, the organization stores and uses its internal knowledge is intimately related to its survival and development [54]. Knowledge-based view emphasizes the integration and learning of knowledge within the organization [51, 55], we not only pay attention to the integration of heterogeneous knowledge from different national boundaries, but also pay attention to the process of enterprises absorbing overseas knowledge with the help of local environmental factors. This is the enrichment and extension of the existing knowledge-based theory.

Third, our research harmonizes the factors associated with overseas and local R&D. From the perspective of knowledge-based view, we include R&D internationalization, absorptive capacity of parent company, domestic technology alliance, and the intensity of competition in local market in the same framework. The research not only from the perspective of the technology acquisition of the host country, but also the capability development of the home country, regards the local factors as the bridge of the technology acquisition of the host country to explore the innovation effect of R&D internationalization. Compared with the literature separately exploring the research on the effect of R&D internationalization on the innovation of the parent company and the research on the impact of home factors on enterprise innovation, our research is more specific and integrity. It will help us understand the phenomenon of overseas research and development more comprehensively.

Last, we found that different from developed countries [56, 57], in the fierce market competition environment in emerging market, local parent company’s absorptive capacity negatively promoted enterprise innovation, however, domestic technology alliances positively promoted enterprise innovation. This helps us to have a deeper analysis of the two-sided results about the economic transformation of emerging markets and a deeper understanding of the resource-based theory. At the same time, the outcoming helps managers to notice the different effect of local competitive environment to local factors, thus adjust the enterprise strategic decisions in time, this also provides theoretical reference for the person in power to weigh the relationship between market and control.

The remainder of this paper is organized as follows: The second section is a theoretical analysis of the relationship between R&D internationalization and parent company innovation, the mediating effect of domestic technology alliances and the moderating effect of market competition intensity. The third section is a description of the data and methods used. The fourth section is the empirical result analysis. The fifth section is the endogeneity test. The sixth section is the robustness test, and the seventh section presents conclusions.

## 2. Theory and hypotheses development

### 2.1. R&D internationalization and the knowledge-based view

The knowledge-based view treats an enterprise as a collection of knowledge [51, 52, 58]. This view holds that knowledge is the single and most important resource of an enterprise, which ultimately determines its unique ability and competitive advantage [59, 60]. The relative value of enterprise technology and capability comes from the difficulty of imitative or transfer of its inherent knowledge assets, which may come from formal mechanisms, such as patents, or from the complex and ambiguous nature of its intrinsic knowledge [61]. How to acquire heterogeneous imitative tacit knowledge is the key to enterprise innovation. The knowledge that underlies an enterprise’s skills and capabilities is often intertwined with its social, organizational, and historical background, and cannot be separated from these unique networks of relationships. This complicated knowledge has become a source of competitive advantage for enterprises due to its high degree of inimitability [62]. However, due to the constraints of domestic political and economic development, emerging market enterprises can’t completely rely on their own ability to gain international competitive advantages, like those in developed countries. Instead, they have to coordinate the resource they can obtain and use it flexibility to fulfill their innovation assignment [63], so they try their best to acquire such inimitable knowledge, so as to develop their own strength and form a knowledge base. According to the OLI compromise investment theory of Dunning, overseas investment by enterprises in developed countries is mostly based on ownership advantage, location advantage and internalization advantage [64], which is an expansion from the inside to the outside. Different from developed countries seeking investment in overseas markets, the primary purpose of emerging market enterprises’ overseas investment is to obtain technological resources and seek innovative breakthroughs, so as to achieve a springboard leap [1]. R&D internationalization plays a crucial role in the acquisition of obscure and complex technical knowledge, which is a bridge to establish the connection between old knowledge and new knowledge and promote innovation. Specifically, R&D sub-institutions can go deep into the internal innovation system of the host country, acquire knowledge from the external relationship network by building relationship assets, knowledge sharing paths and effective relationship governance mechanisms, promoting the enterprise’s innovation ability by relying on the effective connection of new and old knowledge. Therefore, R&D internationalization is the expansion of the knowledge flow boundary of enterprises. On the one hand, enterprises export the knowledge of their parent companies to R&D internationalization institutions to support their expansion in overseas markets. On the other hand, based on the research and development internationalization center, enterprises obtain knowledge overflow through the interaction with the host country environment, and then absorb, transfer, integrate and innovate the complex and fuzzy knowledge, and combine the old knowledge with the new knowledge to promote the improvement of enterprises’ innovation ability.

### 2.2. R&D internationalization and innovation of the parent company

From the perspective of the knowledge-based view, first of all, enterprises can directly obtain heterogeneous resources through R&D internationalization. In terms of external environment, cultural customs and degree of economic development, the host country is quite different from the home country. This kind of difference often brings about heterogeneous knowledge complementary with the home country, which can make up for the deficiency of the home country’s knowledge system [65], enriching the knowledge storage pool on which innovation depends. Secondly, the enterprise, through R&D internationalization behavior, seems to learn more in-depth heterogeneous knowledge. Since the stakeholder groups of the host country are different from those of the home country, enterprises can gain an organizational learning effect by in-depth contact with the stakeholder groups of the host: Enterprises can obtain and learn the upstream scientific and technological information of the supply chain of the host country through communication with suppliers, universities and research institutions of the host country, so as to promote the transfer of upstream complementary knowledge and improve the innovation ability of enterprises. Meanwhile, enterprises can cooperate with interest groups such as distributors, retailers and customers in the host country, so as to learn new market knowledge and product knowledge, and transform the knowledge into application-oriented innovative products to meet the expanded market demands [66]. Also, enterprises in their home countries can acquire rich international operation experience through decentralized R&D activities around the world. By accumulating international R&D and operation experience, enterprise managers in their home countries can enrich their own knowledge structure, so as to quickly and sensitively distinguish the opportunities and threats faced by enterprises and seize the opportunities of new technological changes [67]. Thirdly, the host country usually has a more perfect institutional system than the home country of emerging market enterprises. Emerging market enterprises can gain legitimacy in the host country through legal isomorphism, normative isomorphism, and cognitive isomorphism, and can better absorb and utilize core knowledge. After entering the host country, to a certain extent, latecomers can reduce or avoid the rent-seeking costs in their home country. The host country has a sound intellectual property protection system, transparent supervision and a standardized capital market to encourage emerging market enterprises to implement innovative measures. Enterprises gain the trust of host countries by embedding in the core knowledge system of host countries, and establish the isomorphism of domestic systems and norms to gain legitimacy [13]. It is beneficial for enterprises to better absorb and utilize core knowledge and thus promote the formation of innovation ability. Therefore, our first hypothesis is proposed as follows:

**Hypothesis 1**. R&D internationalization has a positive effect on the innovation of the parent company.

### 2.3. The mediating effect of domestic technology alliances

It is not easy for latecomers to use foreign advanced knowledge and skills to carry out innovation and catch-up strategies. Therefore, they need to process and integrate the knowledge and technical resources gained from internationalization R&D by combining the resources of domestic technology alliances. Domestic technology alliances mainly refer to the alliances formed by cooperative R&D activities between enterprises and domestic stakeholders (such as domestic suppliers, distributors, competitors and universities).

First of all, R&D internationalization promotes the formation of domestic technology alliances of latecomers. Compared with developed countries, the main purpose of latecomers’ overseas investment is to seek strategic assets. Close to the source of innovation, they are rapidly leapfrogging the key knowledge assets of companies in the developed world. The general idea of innovation and technological capacity building is to quickly acquire and adopt advanced foreign technologies and management skills [1]. But this kind of knowledge is usually hard coded, tacit, complex and socially embedded, and in order to acquire technology overflow and cultivate technology learning ability, enterprises need to establish a strong internal knowledge base to identify and judge this knowledge [68, 69], by combining differentiation of international advanced knowledge to make up for their own weak link of knowledge innovation [70, 71]. Therefore, emerging market enterprises tend to form close R&D alliances with domestic suppliers, distributors, customers and even competitors to absorb, integrate and transform the knowledge acquired from R&D internationalization [72, 73], so as to enhance their own R&D strengths. Due to the unity of cultural practices, jet lag and language barriers, trust and loyalty between domestic technology alliance members is stronger, and the members can directly, through face-to-face communication, meetings and other communication forms, obtain knowledge. Relying on more frequent communication to improve the learning ability and technology construction ability of enterprises, technology alliances can improve the quality of enterprise knowledge generation and transmission, help enterprises to absorb the knowledge obtained from R&D internationalization [74]. Secondly, it is easier for enterprises to form economies of scale and economies of scope through domestic technology alliances, so as to reduce the cost of innovation and promote innovation. Economies of scale refers to the phenomenon that enterprises can jointly use the same parts of materials, manpower and finance to reduce production costs and improve production efficiency. As some R&D resources, such as laboratories and R&D equipment, are indivisible, it is more effective for enterprises to make full use of indivisible assets than to invest in various equipment in scattered and small-scale R&D sites [75]. Therefore, it is beneficial to save costs and promote innovation if the parent company of an emerging economy forms technology alliances at home, such as the merging of scientific and technological departments among the same industry or scientific research cooperation. Economies of scope emphasizes that enterprises in different industries and relevant stakeholders can obtain benefits due to the spillover and sharing of resources. Due to the similarity of the economic environment and institutional system faced by domestic technology alliance enterprises, compared with overseas technology alliances, it is easier for domestic technology alliance enterprises to make use of scopes of economy to obtain profits. Enterprises usually set up R&D institutions within the geographical scope of universities, research institutes, domestic government institutions, suppliers and other institutions, so as to gain diversified knowledge and technology spillover in different fields [76, 77], through common technology platforms and proprietary equipment to synergy with the domestic technology alliances [78], so as to obtain the knowledge and technical supplement to promote innovation [73]. In view of this, this paper proposes:

**Hypothesis 2**. R&D internationalization has a positive impact on domestic technology alliances, while domestic technology alliances have a positive impact on the parent company’s technology innovation. Domestic technology alliances have a mediating effect between R&D internationalization and the parent company’s innovation.

### 2.4. The mediating effect of absorption capacity

Absorptive capacity refers to the ability of an enterprise to recognize the value of new external information and absorb it and apply it to business terminals. Zahra and George divide absorptive capacity into potential and actual capacity according to their different effects on competitive advantage [79]. Potential absorption ability includes knowledge acquisition ability and knowledge digestion ability, while actual absorption ability includes knowledge transformation and integration ability and knowledge utilization ability [68]. For multinational enterprises engaged in R&D internationalization, only the advanced technical knowledge acquired through R&D internationalization effectively recognized and absorbed can promote domestic R&D, thus promoting the enterprises’ innovation ability. Kotabe et al. took transnational enterprises in emerging economies as research objects and showed that enterprises must have the absorptive capacity to integrate the knowledge and skill they seek in foreign countries so that international investment in R&D can achieve better innovation performance [80].

First of all, strong absorption capacity can help emerging market enterprises identify the innovation environment and reduce the risk associated with R&D [81]. For example, information intermediaries are key elements of the innovation environment, which reduce transaction costs and promote the dissemination and adoption of information. Although host markets with strong institutions provide high-quality intermediary services, latecomers with strong absorptive capacity can better perceive and even take advantage of such services. R&D internationalization enterprises with high absorptive capacity can quickly identify domestic institutional customs and norms, so as to better cope with institutional pressure, reduce uncertainty, and improve the legitimacy and possibility of their survival in the new environment [41]. On the contrary, companies with insufficient absorption capacity have poor response and utilization ability to the environmental conditions of the host country [82], and are unable to effectively identify and utilize the technological environment and institutional environment of the host country. Secondly, knowledge is the most important resource for product innovation performance, and acquiring new external knowledge is particularly difficult [83]. R&D internationalization enterprises with strong absorptive capacity can realize the gaps in the technology field more quickly and integrate the new external knowledge acquired. Companies with strong absorptive ability will actively search for overseas technical knowledge that has a certain technical gap from their home country through their home-based augmenting strategy [9]. They will merge and acquire complementary knowledge assets, absorb diversified and different knowledge assets, promote the overflow, dispersion and integration of external knowledge, and improve the company’s ability to combine knowledge in different fields and establish connections by learning and integrating the knowledge acquired in the host country [68]. Thirdly, enterprises with strong absorptive capacity can establish long-term and stable cooperative relations with R&D internationalization projects between partners, and better transform knowledge into business results. Arora and Gambardella found that companies with better absorptive capacity had more options, and they often established privileged relationships with a few valuable partners [84]. Fewer and more valuable R&D partners can interact with R&D internationalized subsidiaries to a greater extent, promoting a common understanding of collaborative R&D activities among cooperative stakeholders, leading to the consistency of strategic interests to transform knowledge into business results [85]. This motivation promotes both parties to adjust R&D strategies in a timely manner in the process of task flow and coordination, which increases the learning benefits in the cooperation process and indirectly improves the internationalization benefits of R&D. In view of this, this paper proposes:

**Hypothesis 3**. R&D internationalization promotes innovation of the parent company through absorptive capacity, which acts to mediate between R&D internationalization and innovation of the parent company.

### 2.5. Moderating effect of market competition on domestic technology alliances

In economics, the alliance is formed by two or more business entities which share common strategic interest, under the condition of the fierce market competition, in order to share the market, common use of resources and other strategic objectives, the alliance form a loose cooperation mode through a variety of agreements and contracts, which has complementary advantages and is superior of risk-sharing [86, 87]. With the accelerating global economic integration and the increasingly fierce market competition, scientific and technological innovation is gradually replacing the traditional production factors such as labor, capital and land to become the most important core resources and key elements of enterprise development, and scientific and technological innovation has become the fundamental source for enterprises to obtain competitive advantages for sustainable development [56, 57], It is under this background that technology alliance is formed [88].

Technology alliance is the result of enterprise’s strategic resource demand and social resource opportunity [89–91]. As the market competition becomes more and more fierce, enterprises will choose to carry out technical cooperation with more domestic stakeholders to maintain their market share. As the markets of emerging economies open up gradually, a large number of multinational companies continue to flood into the domestic market, while domestic private enterprises increase rapidly [92, 93], the resources available to an individual enterprise tend to decrease [67, 94]. When resources in an industry are readily available, firms’ willingness to form technology alliances will be greatly reduced, while with the fierce market competition and increasingly difficult access to scarce resources, enterprises tend to choose cooperation to achieve common profits [95]. Therefore, under the fierce market competition, enterprises are more likely to form technological strategic alliances [96]. Grant believed that technology alliances could integrate complementary resources among enterprises more effectively and exploit new market opportunities more accurately [97]. Lambe and Spekman found that if an enterprise could not fully obtain the resources necessary for its development from its own internal sources, in order to achieve its goals, the enterprise could only obtain resources by exchanging them with organizations that had such resources [98]. Specifically, the role of local market competition in promoting the relationship between the innovation of the parent company and the domestic technology alliance is mainly reflected in the following aspects:

First, due to the increasingly fierce competition in the market, the number of technical personnel and equipment to maintain the technical advantages of their products are constantly rising. Through the technology alliance, the enterprise can obtain the necessary technology development equipment and expert group. moreover, the enterprises that participate in the technology alliance often have carried on the development to a certain extent in the technical product project [99, 100]. The technology alliance gathers the development achievements of these enterprises and makes the development achievements marketable by integrating the technological advantages of each enterprise [101], accelerates the speed of new products entering the market, thus promoting the innovation performance of enterprises. Second, in the fierce market competition, in order to reduce innovation risks, enterprises have to seize the market opportunities in the short term to gain competitive advantages [102]. With the acceleration of product upgrading, the risk of developing new products is also increasing. Fierce market competition means that in a relatively short period of time, enterprises must be more efficient in product R&D, design and meet the market demand. At the same time, if the products cannot meet the will of consumers after entering the market, they are likely to be quickly replaced by some more cost-effective products developed by other enterprises [103, 104]. Once an enterprise falls behind its competitors in the development of new products and the marketing channels, a huge amount of development investment will go to waste, which will bring a huge blow to the development of the enterprise. In the fierce market competition, such sunk costs will undoubtedly make the innovation of the enterprise worse. Therefore, when the competition in the local market becomes more and more fierce, in order to reduce innovation risks, it is easier for enterprises to form technological alliances with competitors, universities or research institutes and other relevant stakeholders to jointly bear innovation risks, so as to promote enterprise innovation [105]. In view of this, this paper proposes:

**Hypothesis 4**. Market competition enhances the promotion effect of domestic technology alliances on technological innovation of the parent company.

### 2.6. The moderating effect of market competition on absorptive capacity

Xia et al analyzed the influence of absorptive capacity on the correlation between knowledge transfer and independent innovation, and concluded that based on different degrees of absorptive capacity, the recipient would have different effects on the application of the transferred knowledge and promote independent innovation to different degrees [106]. Companies with high absorptive capacity tend to be more aware of opportunities and information in the market, and more able to digest and integrate tacit and unteachable knowledge [68], but in the fierce market competition for emerging market enterprise, there is something different.

First, in China, the relationship between government intervention in the economy and market economy is substitutional. Intense market competition means more economic liberalization, and smaller space for government regulation. The Chinese government regards government intervention and regulation of the economy as an effective means of economic development [42]. The government can encourage the cooperation between industry, university and research institute through the establishment of innovation network [38], so as to reduce innovation risk and encourage enterprises to improve innovation performance by relying on their own absorptive capacity [45]. For example, a strict property rights system can help enterprises better protect the internal knowledge they use and improve their innovation performance [13, 107]. Many small, micro and even medium-sized enterprises in China account for more than 60 percent of the country’s enterprises, most of them developed gradually with the support of government policies. For example, the Chinese government’s tax on small and micro technology-based enterprises is as low as 13% (compared with 17% for ordinary enterprises). In some regions, technology enterprises are encouraged by policies such as support for R&D expenses, subsidy for unfinished projects, subsidy for loan interest, subsidy for intellectual property application expenses, award for scientific and technological talents, and priority for national project application. These promotion policies encourage enterprises to invest in R&D activities, which is the basis for the increase of absorptive capacity [68]. Fierce competition in the region means that the government’s control power is reduced, in this case, the enterprise ’s absorptive ability cannot get effective protection and support, so the innovation performance of the small and medium-sized technology enterprise may be inhibited.

Second, absorptive capacity can promote both incremental innovation and explorative innovation [108], incremental innovation is small changes and modifications to products and technologies, while explorative innovation is a major departure from a company’s existing capabilities that forms the basis for entirely new products and services [109, 110]. Under the fierce market competition, explorative innovation is more conducive to promoting the innovation performance of the parent company [42, 111]. As the market competition becomes more and more fierce, enterprises tend to adopt explorative innovation to seize the market opportunity, which means that enterprises need to invest more innovation resources than usual. However, fierce market competition also means that enterprises need to develop diversified products, diversified markets and cost-effective performance of products, which will cause more resource consumption [102], as a result it’s not conducive for enterprises to concentrate resources for explorative innovation.

Third, the energy of enterprise managers is limited [112–115], therefore, the fierce market competition may make the enterprise explore diversified products, diversified markets and recruit more talents, which will distract the managers’ energy in R&D investment and the cultivation of absorptive capacity of the enterprise, thus it will not contribute to the positive effect of absorptive capacity on the innovation of the parent company. In view of this, this paper proposes:

**Hypothesis 5**. The promotion effect of the absorptive capacity on the technological innovation of the parent company is weakened by increased market competition.

**Fig 1:**
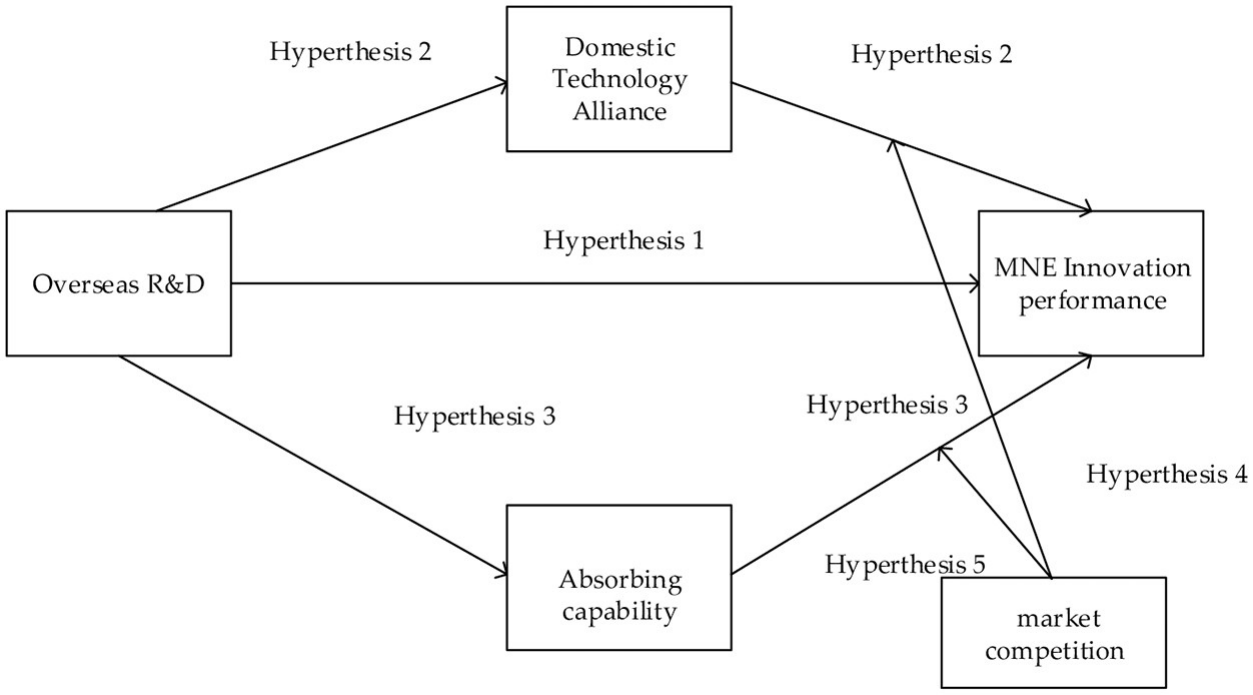
Logic frame figure.

The conceptual framework is described based on the above assumptions, as shown in Fig 1:

## 3. Methodology and data

### 3.1. Models and methodology

In this paper, first of all, the mediating effect is adopted to analyze the data, and the effect of R&D internationalization on the parent company’s innovation is generated through the mediating effect of domestic technology alliances and absorption capacity. According to the analytical steps of Baron and Kenny on the mediating effect [116], the following five models are established successively.

$$patent_{it}=\alpha_{0}+\alpha_{1}ovrd_{it}+\alpha_{2}controls_{it}+\lambda_{i}+\gamma_{a}+\mu_{t}+\xi_{it}$$ (1)

$$doteal_{it}=\beta_{0}+\beta_{1}ovrd_{it}+\beta_{2}controls_{it}+\lambda_{i}+\gamma_{a}+\mu_{t}+\xi_{it}$$ (2)

$$patent_{it}=\delta_{0}+\delta_{1}ovrd_{it}+\delta_{2}doteal_{it}+\delta_{3}controls_{it}+\lambda_{i}+\gamma_{a}+\mu_{t}+\xi_{it}$$ (3)

$$absorp_{it}=\eta_{0}+\eta_{1}ovrd_{it}+\eta_{2}controls_{it}+\lambda_{i}+\gamma_{a}+\mu_{t}+\xi_{it}$$ (4)

$$patent_{it}=\kappa_{0}+\kappa_{1}ovrd_{it}+\kappa_{2}absorp_{it}+\kappa_{2}controls_{it}+\lambda_{i}+\gamma_{a}+\mu_{t}+\xi_{it}$$ (5)

*patent*_*i*t_ refers to the explained variable which stands for the innovation of the parent company, *ovrd*_*it*_ stands for the R&D internationalization of the explanatory variable, *dord*_*it*_ represents the domestic technology alliances, *absorp*_*it*_ represents the absorptive capacity of the mediating variable. *controls*_*t*_ represents all control variables, *i* represents the industry of the company, *a* represents the region (province) where the company is located, *t* represents the time., *λ*_*i*_, *γ*_*a*_, *μ*_*t*_ represents the industry effect, regional effect and time effect of the company, and *ξ*_*it*_ is the residual term.

Eqs (1), (2) and (3) are the test of the mediating effect of domestic technology alliances: Eq (1) is the influence of the core independent variable R&D internationalization on innovation of the parent company, and *α*_1_ is the overall effect of the core independent variable on the dependent variable. Eq (2) is the promotion effect of R&D internationalization on domestic technology alliances and *β*_1_ stands for the influence effect of R&D internationalization on domestic technology alliances. Eq (3) is the effect of R&D internationalization and domestic technology alliances on the parent company’s innovation, and *δ*_1_ is the direct effect of the parent company’s innovation, when *δ*_1_ is significant, it represents that domestic technology alliances have a partial intermediary effect to the R&D internationalization, when *δ*_1_ is not significant, it means domestic technology alliances play a full mediation role, which means the influence of R&D internationalization on parent company innovation comes totally through domestic technology alliances. *δ*_2_ * *β*_1_ is the indirect effect of R&D internationalization through mediating variables. Eqs (1), (4) and (5) are the test of the mediating effect of absorption capacity: Eq (4) is the promotion effect of R&D internationalization on absorption capacity; Eq (5) is the joint influence of R&D internationalization and absorption capacity on parent company innovation; *κ*_2_ * *η*_1_ is the indirect effect of R&D internationalization through absorption capacity.

At the same time, this paper analyzes the moderating role of market competition, and establishes a regulated mediation model based on the research of Muller [117]. The specific equations and test steps are as follows:

$$patent_{it}=\nu_{0}+\nu_{1}ovrd_{it}+\nu_{2}comp_{it}+\nu_{3}ovrd_{it}*comp_{it}+\nu_{4}controls_{it}+\lambda_{i}+\gamma_{a}+\mu_{t}+\xi_{it}$$ (6)

$$doteal_{it}=\rho_{0}+\rho_{1}ovrd_{it}+\rho_{2}comp_{it}+\rho_{3}ovrd_{it}*comp_{it}+\rho_{4}controls_{it}+\lambda_{i}+\gamma_{a}+\mu_{t}+\xi_{it}$$ (7)

$$patent_{it}=\theta_{0}+\theta_{1}ovrd_{it}+\theta_{2}comp_{it}+\theta_{3}ovrd_{it}*comp_{it}+\theta_{4}doteal_{it}+\theta_{5}doteal_{it}*comp_{it}+\theta_{6}controls_{it}+\lambda_{i}+\gamma_{a}+\mu_{t}+\xi_{it}$$ (8)

$$patent_{it}=\omega_{0}+\omega_{1}ovrd_{it}+\omega_{2}comp_{it}+\omega_{3}ovrd_{it}*comp_{it}+\omega_{4}absorp_{it}+\omega_{5}comp_{it}*absorp_{it}+\omega_{6}controls_{it}+\lambda_{i}+\gamma_{a}+\mu_{t}+\xi_{it}$$ (9)

$$absorp_{it}=\kappa_{0}+\kappa_{1}ovrd_{it}+k_{2}comp_{it}+\kappa_{3}ovrd_{it}*comp_{it}+\kappa_{4}controls_{it}+\lambda_{i}+\gamma_{a}+\mu_{t}+\xi_{it}$$ (10)

Eqs (6), (7) and (8) are tests of the moderating effect of competitiveness on domestic technology alliances, while Eqs (6), (9) and (10) are tests of the moderating effect of competitiveness on absorptive capacity. In the first step as shown in Eq (6), we inspect the significance of *ν*_3_, in the second step as shown in Eq (7), the test is whether *ρ*_2_ or *ρ*_3_ is significant, and in the third step as shown in Eq (8), we test the significance of the coefficient *θ*_3_ or *θ*_5_. If *ρ*_3_ and *θ*_3_ are significant at the same time, the market competition moderates the relationship between the R&D internationalization and domestic technology alliances. If *ρ*_2_ and *θ*_5_ are significant at the same time, the market competition moderates the relationship between the technology alliances and innovative performance of the parent company. The test procedure of Eqs (9) and (10) is the same as that of Eqs (7) and (8).

Since the number of patent applications and other data in different years are different, we follow Jaffe & Trajtenberg to control the annual variable to reduce the systematic year-to-year difference caused by "truncation bias" [118]. Similarly, fixed effects help to overcome individual differences. Since the dependent variable parent company’s innovation is measured by counting data, we adopt a high-dimensional Poisson fixed effects model to test all the models. The high-dimensional Poisson fixed-effect model does not need to satisfy the assumption that Poisson regression requires sample mean to be equal to variance [119], and the dependent variable is not limited to counting data [120]. Therefore, the model with domestic technology alliances as the dependent variable can still be tested.

### 3.2. Variables selection and measurement

According to the industry classification of China Securities Regulatory Commission in 2001, we select some industries with frequent R&D internationalization activities, which include the computer application service (G87), communication services (G85), computer and related equipment manufacturing industry (G83), communication and related equipment manufacturing industry (G81), pharmaceutical manufacturing (C81), special equipment manufacturing (C73), electronic components manufacturing (C51), and chemical raw materials and chemical manufacturing (C43). Because these enterprises in the industries which spend more resource and pay more effort on RD internationalization while other industries may have only a few RD activities abroad, the selection of these samples can maximize our sample size. We select the above eight industries as the sample base. In the first step we exclude ST enterprise sample. ^1^ST shares refer to listed companies in the territory of two consecutive years of losses, so the stock need to be special treated. In the second step, we remove the samples of subsidiaries established in tax havens such as "Cayman Islands", " Virgin Islands", "Bermuda" and "United Arab Emirates".

In the third step, we remove samples with unclear business scope and incomplete data disclosure. In the fourth step, we remove the missing samples and outlier samples. In the fifth step, we remove the samples whose business scope in the six years from 2012 to 2017 does not include the words "technology development", "scientific research", "research", "research and development" and "science and technology". Finally, we obtain the balance panel data of 185 samples per year from 2012 to 2017, and a total of 1,110 samples were obtained.

#### 3.2.1. Dependent variable: Innovation

At present, the measurement of innovation of the enterprise mainly includes two ways: one is to take the added value of new products as the measurement standard, which can reflect the commercial value of innovation activities, but there is no unified standard for the division of new products, so it has great deficiencies in application. Another is the number of patents as a measure, including patent application, patent license number, number of patent references and valid patents. Because the quantity of patent applications is associated directly with the enterprise’s innovation activities, it reflects the degree of enterprise innovation [121], and directly promotes the improvement of enterprise asset value [122]. We measure the innovation of the R&D international parent company by the total number of patent applications, and the relevant data are obtained from the Guotaian (CSMAR) database and the company’s annual reports.

#### 3.2.2. Independent variable: Internationalization of R&D

At present, R&D internationalization is mainly measured in the following ways: (1) R&D internationalization is measured in the form of a binary dummy variable, which is assigned as "1" for those with R&D internationalization and "0" for those without [22], but this measurement method is relatively rough. (2) The ratio of R&D internationalization expenditure to total expenditure is used to indicate the depth of R&D internationalization, but the sample data is only updated to 2015. (3) The patent application volume of each country where the patent inventor is located [26]. This method has not been provided with detailed information in the database of China at present, so the data is difficult to obtain. (4) By measuring the sum of different regions of the number of patents applied for by the parent company’s overseas subsidiaries [34]. However, some patents are directly applied for by the parent company, so there will be some measurement deviation. Finally, according to Hsu et al, the proportion of R&D internationalization subsidiaries in the total subsidiaries is taken into account, which can better reflect the depth of R&D internationalization [33]. We manually calculate the number of samples each year containing the words "technology development", "scientific research", "research", "research and development" and "science and technology", and calculate the total number of overseas subsidiaries of each company and calculate the ratio of the two. The relevant data are from the List of Overseas Investment published by the Ministry of Commerce and the Guotaian (CSMAR) database.

#### 3.2.3. Mediating variable: Domestic technology alliances

We search on the Internet by each company’s stock code, announcement of public information, company annual reports, company news, and related party transactions. We manually record the number of times each company conducts technical cooperation, accepts technology transfers and management services, conducts joint research and development, and conducts joint technology development with domestic suppliers, universities, and companies in the same industry and companies in other industries each year. If the number is zero, it is recorded as 0. If there is no change compared with the previous year, the data of the previous year is still used, to maintain comparability between samples, we used a relative indicator, which is the number of technology alliance divided by the total number of company collaborations in that year.

Absorption capacity: R&D costs will affect the extent to which enterprises use external knowledge [68, 123]. Many scholars measure absorption capacity by R&D expenditure [33, 124], such as R&D spending accounting for the proportion of revenue, R&D spending accounting for the proportion of total assets. However, due to the influence of the natural environment, economic situation and industry situation, the business situation is unstable. In this paper, the proportion of R&D expenses in the total assets at the beginning is used to measure the absorptive capacity, and relevant data are obtained from the Ruisi (RESSET) database and company annual reports.

#### 3.2.4. Moderating variables

Market competition: market competition measures the intensity of market competition in an enterprise’s domestic sphere. According to the methods used by Glaeser et al and Gao [125, 126], we estimate the competition intensity of domestic regional markets. We first calculate the industry competition index of each region as follows:

$$c_{ij}=\frac{\left(n_{ij}/r_{ij}\right)}{\left(n_{j}/r_{j}\right)}$$ (11)

*n*_*ij*_ represents the total number of enterprises owned in industry *j* in the province region *i*; *r*_*ij*_ represents the total sales volume of enterprises in industry *j* in province *i; n*_*j*_ represents the number of enterprises in sector j at the national level; *r*_*j*_ represents the national sales revenue of the *j* sector. The high value of the sector-region portfolio index indicates that there are more active enterprises in this sector-region than there are nationwide, and the degree of competition is more intense. We average the industry index values in each region to generate the overall competition index for each company in each region (*n*_*i*_ represents the number of industries owned by each region):

$$comp_{i}=\frac{1}{n_{i}}\sum_{j=1}^{n}c_{ij}$$ (12)

the relevant data are obtained from the Industrial enterprise database and the National Bureau of Statistics.

#### 3.2.5. Control variables

1. Company age: Older companies tend to have rich operating experience, a more standardized company system, articles of association and reasonable organizational form. We measure the difference between the company’s registration date and the year of measurement [34, 127], and related data are obtained from Guotaian (CSMAR) database and enterprise annual reports.
2. Company size: Larger companies often have a better ability to develop internal knowledge, and absorb and combine external knowledge for innovation [28], and at the same time are more likely to develop economies of scale and save on costs of innovation. We use the logarithm of the total assets at the end of each year to measure the size of the enterprise. The relevant data are from Guotaian (CSMAR) database and the annual reports of the enterprise.
3. Experience in enterprise internationalization: Enterprises with rich experience in internationalization are more familiar with overseas institutional rules and economic environments, so they can respond to sudden changes more sensitively, which is conducive to creating a good environmental foundation for enterprise innovation [33]. For some enterprises lacking experience in internationalization, the technical complexity, market uncertainty and high risk in the international market increase the difficulty of enterprise innovation. In this paper, the internationalization experience of enterprises is measured by observing the total number of overseas companies in the previous year of the parent company. Relevant data are obtained from the Guotaian (CSMAR) database and annual reports of enterprises.
4. Return on Equity (ROE): Based on the practice of Yuan [128], this paper adopts return on equity (ROE) as the control variable in the model. This index reflects the income level of shareholders’ equity, which is used to measure the efficiency of the company using its own capital. Return on equity is equal to net income after taxes divided by total assets at the beginning. The higher the return on equity is, the stronger the enterprise’s ability to obtain net income with its own capital is, which lays a foundation for innovation. The relevant data are obtained from Ruisi (RESSET) database and corporate annual reports.
5. TobinQ: TobinQ is the ratio of the market value of an enterprise to the replacement cost of capital. Its economic meaning is to compare whether the market value of enterprises as economic subjects is greater than the cost of capital that brings cash flow to enterprises, which is an important factor for enterprises to decide investments [129]. The higher the TobinQ is, the more inclined the enterprise is to invest and the stronger the motivation for innovation is. Relevant data are obtained from Ruisi (RESSET) database and corporate annual reports.
6. Enterprise cash flow: Cash flow is an important indicator to measure whether an enterprise has a good operating condition, whether it has enough cash to repay debts and the liquidity of assets. Sufficient cash flow means that an enterprise has a stable innovation environment and abundant innovation funds. We measure cash flow by the level of cash held at the beginning adjusted for total assets as shown in the balance sheet. The relevant data are obtained from Ruisi (RESSET) database and corporate annual reports.
7. Enterprise tax intensity: In this paper, enterprise tax intensity is taken as a control variable of the model. Tax intensity reflects the proportion of enterprise tax in main business income. High tax intensity means that enterprises will lose part of the funds used to develop their own business, which will have a certain impact on innovation. We take the tax paid by the enterprise divided by the main business income as the index to measure the tax intensity of the enterprise. The relevant data are obtained from the Ruisi (RESSET) database and the annual reports of the enterprise.
8. Market institutional quality: In this paper, we adopt “China’s Marketization Index—A 2019 Report on the Relative Marketization Process of Various Regions” by Fan Gang and Wang Xiaolu to measure the institutional quality of the regions where enterprises are located [130]. It is based on 23 sub-indicators from five aspects: the relationship between the government and the market, the development of the non-state-owned economy, the development of the product market, the development of the factor market, the development of the market intermediary organization and the legal system environment. The index is constructed by using Principal Component Analysis as the basic measurement method. However, due to the slow update of this index, the earliest data is only updated to 2016. Therefore, we draw lessons from the practice of Ma et al [131], and take the average growth rate of the marketization index over the years as the forecast basis for 2017 to predict the market system quality in 2017.
9. Regional dummy variable: Due to the differences in infrastructure construction, history and culture, policy priorities, and economic development in different regions, we also control the province where each parent company is located as the regional control variable.
10. Industry dummy variable: We also control the industry variable due to the different development foundation, development stage and demand degree of technology and innovation in each industry. According to industry level ii codes and industry names, the industries are divided into 59 industries. The industry classification codes and standards are obtained from the Ruisi (RESSET) database.

All variables are described and sourced in Table 1.

**Table 1:** Variable source and definition table.

Variable types	symbol	description	observations	mean	sd	minimum	maximum
Explained variable	patent	Innovation performance	1,110	190.454	404.659	2.000	2616.000
explanatory variable	ovrd	R&D internationalization	1,110	0.323	0.380	0.000	1.000
mediating variables	doteal	Domestic Technology Alliance	1,110	2.310	2.603	0.000	10.000
	absorp	Absorptive capacity	1,110	3.751	3.218	0.306	21.439
moderating variable	comp	Market competition	1,110	0.977	0.350	0.474	1.886
Control variables	size	Enterprise size (logarithm)	1,110	22.287	1.182	20.143	25.715
	age	Enterprise age	1,110	14.978	5.373	6.000	37.000
	exper	Overseas Investment experience	1,110	3.929	3.482	1.000	21.000
	roe	Return on equity	1,110	0.081	0.138	-2.179	1.611
	tobinq	The market value of the business divided by the replacement cost of the assets	1,110	2.625	2.518	0.113	33.475
	cash	Cash flow (logarithm)	1,110	20.368	1.219	17.536	24.107
	revenue	The intensity of tax	1,110	0.015	0.019	-0.207	0.145
	market	Institutional quality	1,110	0.708	0.110	0.284	0.836
Instrumental variable	alls	Overseas investment scale of companies in other industries other than the parent company (logarithm)	1,110	6.489	0.253	5.852	6.958
	RDS	The mean overseas R&D subsidies in other industries other than the parent company	1,110	2.897	3.151	0.000	19.000

## 4. Empirical results and analysis

### 4.1 Descriptive statistics and correlation analysis

From the variable relationship description table (Table 2), we can see that R&D internationalization has a significant positive impact on the innovation of the parent company (P<0.01), and there is a significant correlation between R&D internationalization and domestic technology alliances (P<0.01), which preliminarily confirms part of our speculation. However, the correlation between domestic technology alliances, absorptive capacity and the innovation of the parent company is not significant, which needs to be further analyzed and demonstrated. Pearson correlation coefficients between the variables are less than 0.5, in the analysis of variance inflation factor. After mean centralization of related variables in the interaction item model, the VIF values of each model are shown in Table 3. The VIF value of each model is between 1.18 and 1.20, which is lower than the usual threshold value of 5, confirming that there is no multicollinearity between variables.

**Table 2:** Variable relationship description table.

	**patent**	**ovrd**	**doteal**	**absorp**	**comp**	**size**	**Age**	**exper**	**roe**	**tobinq**	**cash**	**revenue**	**market**
patent	1.0000												
ovrd	0.126***	1.0000											
doteal	0.023	0.090***	0.111***										
absorp	0.001	-0.069**	-0.084***	1.0000									
comp	-0.045	-0.042	-0.080***	-0.144***	1.0000								
size	0.564***	-0.008	-0.016	-0.132***	-0.148***	1.0000							
age	0.094***	-0.166***	0.047	-0.055**	-0.064**	0.239***	1.0000						
exper	0.299***	0.081***	0.124***	-0.021	-0.028	0.430***	0.125***	1.0000					
roe	0.111***	0.027	0.109***	0.011	-0.007	0.114***	0.011	0.032	1.0000				
tobinq	-0.204***	0.020	-0.012	0.275***	0.275***	-0.381***	-0.111***	-0.118***	0.205***	1.0000			
cash	0.148***	0.012	-0.010	0.006	0.009	0.279***	0.197***	0.139***	0.006	-0.091***	1.0000		
revenue	-0.117***	-0.042	-0.005	0.018	0.007	-0.034	-0.029	-0.070**	0.143***	0.144***	0.001	1.0000	
market	-0.052*	0.020	-0.012	0.135***	0.135***	-0.011	-0.039	0.056*	0.033	0.102***	0.113***	0.03	1.0000

*** p<0.01,

** p<0.05,

* p<0.1

**Table 3:** Mediating effect test and moderated mediating effect test.

VARIABLES	m1	m2	m3	m4	m5	m6	m7	m8	m9	m10	m11
	patent	patent	doteal	patent	absorp	patent	patent	doteal	patent	absorp	patent
ovrd		0.479***	0.707***	0.349***	0.160**	0.441***	0.250***	0.360***	0.217***	0.095**	0.231***
		(0.107)	(0.092)	(0.106)	(0.065)	(0.110)	(0.039)	(0.069)	(0.040)	(0.046)	(0.038)
doteal				0.058***					0.059***		
				(0.015)					(0.015)		
absorp						0.056***					0.053***
						(0.015)					(0.015)
comp							0.498**	0.212	0.535**	0.641*	0.452*
							(0.247)	(0.366)	(0.241)	(0.361)	(0.251)
ovrd*comp							-0.401***	-0.255**	-0.408***	0.006	-0.340***
							(0.098)	(0.100)	(0.106)	(0.073)	(0.095)
doteal*comp									0.039***		
									(0.014)		
absorp*comp											-0.012*
											(0.007)
size	0.675***	0.680***	-0.074**	0.683***	-0.052*	0.695***	0.667***	-0.108***	0.669***	-0.062**	0.683***
	(0.044)	(0.040)	(0.038)	(0.038)	(0.030)	(0.040)	(0.040)	(0.039)	(0.038)	(0.030)	(0.040)
age	0.017**	0.015**	-0.014*	0.018**	0.005	0.013*	0.018**	-0.011	0.020**	0.005	0.017**
	(0.008)	(0.008)	(0.007)	(0.008)	(0.005)	(0.008)	(0.008)	(0.007)	(0.008)	(0.005)	(0.008)
exper	0.033***	0.041***	0.021**	0.040***	0.000	0.040***	0.044***	0.021**	0.044***	-0.000	0.044***
	(0.009)	(0.008)	(0.010)	(0.007)	(0.007)	(0.008)	(0.008)	(0.010)	(0.008)	(0.007)	(0.008)
roe	0.185***	0.162***	0.109***	0.143***	-0.016	0.175***	0.160***	0.126***	0.143***	-0.014	0.177***
	(0.055)	(0.053)	(0.036)	(0.053)	(0.029)	(0.053)	(0.052)	(0.037)	(0.051)	(0.030)	(0.051)
tobinq	-0.032	-0.031	0.007	-0.034	0.050***	-0.050*	-0.032	0.005	-0.034	0.049***	-0.048*
	(0.033)	(0.031)	(0.014)	(0.032)	(0.009)	(0.029)	(0.031)	(0.015)	(0.031)	(0.009)	(0.029)
cash	-0.028	-0.032	0.016	-0.028	0.003	-0.033	-0.040	0.005	-0.033	0.001	-0.040
	(0.035)	(0.032)	(0.029)	(0.030)	(0.027)	(0.034)	(0.033)	(0.029)	(0.030)	(0.026)	(0.034)
revenue	-0.133**	-0.130**	-0.034	-0.122**	-0.098**	-0.103**	-0.126**	-0.030	-0.122**	-0.098**	-0.106**
	(0.053)	(0.052)	(0.043)	(0.052)	(0.040)	(0.052)	(0.051)	(0.043)	(0.052)	(0.040)	(0.052)
market	-0.117	-0.082	-0.119	-0.056	0.079	-0.101	-0.109	-0.116	-0.080	0.079	-0.120
	(0.110)	(0.104)	(0.108)	(0.101)	(0.062)	(0.104)	(0.104)	(0.110)	(0.100)	(0.062)	(0.103)
Constant	-8.780***	-9.285***	3.169**	-9.800***	1.547**	-9.568***	-8.450***	4.324***	-8.904***	1.949**	-8.918***
	(1.133)	(1.114)	(1.276)	(1.094)	(0.786)	(1.118)	(1.086)	(1.286)	(1.060)	(0.835)	(1.093)
Observations	1,110	1,110	1,104	1,110	1,110	1,110	1,110	1,104	1,110	1,110	1,110
Pseudo R2	0.794	0.802	0.174	0.807	0.196	0.806	0.806	0.166	0.811	0.196	0.81
Waldchi2	979.9	1114	101.3	1191	90.88	1136	1169	74.37	1301	90.63	1174
Area FE	YES	YES	YES	YES	YES	YES	YES	YES	YES	YES	YES
Industry FE	YES	YES	YES	YES	YES	YES	YES	YES	YES	YES	YES
Year FE	YES	YES	YES	YES	YES	YES	YES	YES	YES	YES	YES
VIF	1.21	1.20	1.20	1.20	1.20	1.20	1.18	1.18	1.19	1.18	1.20

*** p<0.01,

** p<0.05,

* p<0.1

### 4.2. Mediating effect test and moderated mediating effect test

We first test the mediating effect of domestic technology alliances and absorptive capacity, and then test the moderating effect of market competition on domestic technology alliances and absorptive capacity, as shown in Table 3.

#### 4.2.1 Mediating effect test of domestic technology alliances

Model 1 is the test of the promotion effect of control variables on the innovation of the parent company; Models 2, 3 and 4 are the test of the mediating effect of domestic technology alliances; Model 2 is the test of the effect of R&D internationalization on the innovation of the parent company; Model 3 tests the effect of domestic technology alliances on the parent company’s innovation. Model 4 examines the relationship between domestic technology alliances, R&D internationalization and parent company innovation when controlling for other variables. Like the mediating effect test of domestic technology alliances, models 2, 5 and 6 are mediating effect tests of absorptive capacity.

Model 1 shows that the controlling variables, enterprise size, enterprise age, enterprise overseas investment experience and ROE have significant positive correlation with the parent company’s innovation (*β* = 0.6735, *p* < 0.01; *β* = 0.0166, *p* < 0.05; *β* = 0.0332, *p* < 0.01; *β* = 0.1851, *p* < 0.01;). The effect of corporate tax intensity on corporate innovation was negatively correlated (*β* = −0.1328, *p* < 0.05). To be specific, large enterprises usually have more sufficient scientific research funds, more abundant human resources and production input factors, so they have stronger anti-risk ability. Lhuillerys & Plistere and Marchivd & Tether also confirmed the positive correlation between enterprise size and innovation [132, 133]. The positive correlation between enterprise age and innovation may be due to the fact that older companies tend to have a more solid hardware foundation, rich operation experience, a standardized company system and broader and stable cooperation networks, which are conducive to innovation [134]. The overseas investment experience of enterprises can help them to have a more comprehensive understanding of the investment market, judge the investment form and investment environment more keenly [135], and thus reduce the risk of innovation failure. The positive effect of equity indicates that the enterprise has a strong ability to obtain net income by using its own capital, thus laying a good capital foundation for enterprise innovation. Tax intensity being negatively significant indicates that high tax intensity occupies the funds used by enterprises to develop their own business and has a negative impact on innovation. The negative but not significant influence of the degree of market opening up on the innovation of enterprises indicates that the open market environment may lead to more fierce market competition among enterprises [136, 137], which causes enterprises to pay more attention to short-term business performance, thus ignoring the profits brought by long-term innovation investment. Tobin Q has a negative but not significant impact on enterprise innovation, which may be due to the fact that enterprises tend to increase investment but they are not conducive to technological innovation. Diversified investment tends to lead to excessive diversification of enterprise energy and capital, thus resulting in weak innovation. The cash flow of an enterprise is not significant, which maybe means the more sufficient the cash flow of an enterprise is, the more likely managers are to waste cash on inefficient investment projects, which is not beneficial to enterprise innovation [138].

Referring to the method of Baron and Kenny [116], we carry out tests on mediating effects of models 2, 3 and 4. The test results of model 2 show that the internationalization of R&D can significantly promote the innovation performance of the parent company (*β* = 0.4787, *p* < 0.01). This shows that different from the traditional internalization theory of international business [46, 47], the international expansion of emerging market firms is aimed at technology seeking. R&D internationalization plays a crucial role in the innovation catch-up of emerging market enterprises [139]. Model 3 showed that domestic technology alliances have a promoting effect of parent company’s innovation (*β* = 0.7072, *p* < 0.01). Model 4 shows that R&D internationalization and domestic technology alliances have a significant influence on innovation of the parent company (*β* = 0.3487, *p* < 0.01; *β* = 0.0582, *p* < 0.01). According to models 2, 3 and 4, domestic technology alliances play a mediating role in the process of R&D internationalization to promote the innovation of the parent company, meaning hypothesis 2 is supported. It confirms the previous research established that technology alliances often have a positive impact on the technological performance of companies [140, 141]. Technology alliances are often used by companies’ instruments to acquire technological knowledge and to develop new skills that reside within the partnering companies [141–143]. Similarly, in the test results of model 5 and 6, R&D internationalization has a significant promotion effect on absorption capacity (*β* = 0.1604, *p* < 0.01), while R&D internationalization and absorption capacity have a significant promotion effect on the innovation of the parent company (*β* = 0.4412, *p* < 0.01; *β* = 0.0555, *p* < 0.01), meaning hypothesis 3 is supported. This indicates that absorptive capacity is helpful for enterprises to better integrate and digest the acquired external knowledge, and at the same time convert the knowledge into business results [68], it is an important driver of innovation [108].

#### 4.2.2. Test of the moderating effect of absorption capacity and the moderated mediating effect

According to Muller [117], we use the step-by-step test method of the moderating mediation model, and in turn to test the model. Model 7 tests the relationship between R&D internationalization, the interaction terms between R&D internationalization and market competition to innovation, R&D internationalization, and the interaction terms between R&D internationalization and market competition both have significant promoting effects on enterprise innovation (*P*<0.01). Model 8 is a test of the positive effect of R&D internationalization on domestic technology alliances, and the promotion effect of R&D internationalization on domestic technology alliances is extremely significant (*β* = 0.3603, *p* < 0.01). Model 9 is a test of the interaction effect between domestic technology alliances and market competition. The interaction between domestic technology alliances and market competition has a significant positive effect on innovation (*β* = 0.0392, *p* < 0.01), indicating that market competition positively moderates the promoting effect of domestic technology alliances on parent company’s innovation. Therefore Hypothesis 4 is supported, it indicates that fierce market competition enables firms to scan their environment for new windows of opportunities and promising new technologies [105].

Model 10 is a test of the effect of R&D internationalization on absorption capacity, which is significantly positive (*β* = 0.0952, *p* < 0.01). Model 11 is to test the interaction between absorptive capacity and R&D internationalization and its promoting effect on enterprise innovation (*β* = −0.0120, *p* < 0.1), and illustrates the influence of absorptive capacity of technology innovation has a positive role in promoting innovation, but fierce market competition hinders the effect on innovation of the parent company, meaning Hypothesis 5 is supported. This indicates that, different from developed countries, the excessively fierce market competition means the reduction of government regulation. However, in China, moderate government regulation is beneficial to the exertion of enterprises’ absorptive capacity [42]. At the same time fierce market competition may also lead to the diversification of competitive market and product [102, 105], which is not conducive to concentrating resources to promote the development of absorptive capacity of enterprises.

## 5. Endogeneity test

### 5.1. Endogeneity description

In the above empirical models, there may be endogenous problems leading to bias of regression results. First of all, there may be endogenous problems between R&D internationalization and parent company’s innovation: one may be omitted variable bias, that is, some variables are related to R&D internationalization, but due to their difficulty in measurement and concealment these variables are placed in the error term, and the error term is related to R&D internationalization variables and generates endogenous problems. The second is reverse causality, that is, internationalization R&D can promote the innovation of the parent company, while the internationalization of the parent company will in turn promote more R&D internationalization activities. The third is the self-selection of samples: that is, enterprises with more international R&D are those with more R&D activities. These problems may also exist in the model of domestic technology alliances and absorption capacity as dependent variables.

### 5.2. Regression of 2SLS

We select "the scale of overseas investment in other industries other than the parent company’s" and the “mean overseas R&D subsidies in other industries other than the parent company” as the instrumental variable of the core independent variable. We calculate the number of overseas investment subsidiaries of all industries in the sample each year, and classify them according to the industry second-level code. By excluding the number of overseas investment subsidiaries in the industry where a single sample is located, the number of overseas investment subsidiaries in other industries other than the industry where the parent company is located is obtained as a proxy variable of the overseas investment scale of other industries. "The number of overseas investment subsidiaries in other industries other than the parent company" reflects the scale of overseas investment in other industries. They may have a certain competitive relationship with the R&D internationalization subsidiary in the raw material and semi-finished products market, but it has nothing to do with the innovation and absorptive capacity of the parent company and the domestic technology alliances, so as to meet the exogenous of the instrumental variable. The choice of mean overseas R&D subsidies in other industries other than the parent company is based on the similar reason. We use the 2SLS model to further test the model. The specific method is as follows: First, the instrumental variable is used to separate the exogenous part of the core explanatory variable and obtain the fitting value. Second, we use the fitting value to carry on the regression to the dependent variable. We also need to test the exogeneity of weak instrumental variables and instrumental variables. The F value of Kleibergen-Paaprk Wald is much larger than the critical value under the 10% bias, and the F value of the first-stage regression is also much larger than 10, rejecting the hypothesis of weak instrumental variables [144]. To further test the correlation of instrumental variables, the regression results of the first stage are listed, as shown in Table 4. Instrumental variables were significantly correlated with endogenous explanatory variables (P<0.01). Table 5 shows the regression results of 2SLS, excluding the model without endogenous explanatory variables. Models 1, 2 and 3 are the tests of the mediating effect of domestic technology alliances. Model 1, 4 and 5 are the tests of the mediating effect of absorptive capacity. Models 6–10 are the tests of the regulating effect of market competition. All hypotheses are valid.

**Table 4:** Regression results of the first stage.

VARIABLES	m1	m2	m3	m4	m5	m6	m7	m8	m9	m10
	ovrd	ovrd	ovrd	ovrd	ovrd	ovrd	ovrd	ovrd	ovrd	ovrd
alls	-0.073**	-0.073**	-0.071**	-0.073**	-0.070**	-0.076**	-0.076**	-0.075**	-0.076**	-0.073**
	(0.035)	(0.035)	(0.034)	(0.035)	(0.035)	(0.035)	(0.035)	(0.035)	(0.035)	(0.035)
RDS	-0.185***	-0.185***	-0.180***	-0.185***	-0.184***	-0.183***	-0.183***	-0.178***	-0.183***	-0.183***
	(0.006)	(0.006)	(0.006)	(0.006)	(0.006)	(0.006)	(0.006)	(0.006)	(0.006)	(0.006)
Constant	1.269***	1.269***	1.183***	1.269***	1.245***	1.281***	1.281***	1.228***	1.281***	1.264***
	(0.281)	(0.281)	(0.281)	(0.281)	(0.281)	(0.281)	(0.281)	(0.281)	(0.281)	(0.282)
Observations	1,110	1,110	1,110	1,110	1,110	1,110	1,110	1,110	1,110	1,110
R-squared	0.499	0.499	0.503	0.499	0.500	0.501	0.501	0.506	0.501	0.502
F	109.5	109.5	101.1	109.5	99.90	91.64	91.64	79.95	91.64	78.74
Kleibergen-Paaprk	123.778	123.778	112.832	123.778	122.587	121.668	121.668	112.588	121.668	120.644
Controls	YES	YES	YES	YES	YES	YES	YES	YES	YES	YES

*** p<0.01,

** p<0.05,

* p<0.1

**Table 5:** Regression results of 2SLS.

VARIABLES	m1	m2	m3	m4	m5	m6	m7	m8	m9	m10
	patent	doteal	patent	absorp	patent	patent	doteal	patent	absorp	patent
ovrd	0.213***	0.295***	0.185***	0.121***	0.204***	0.211***	0.304***	0.188***	0.106***	0.204***
	(0.029)	(0.035)	(0.031)	(0.025)	(0.030)	(0.029)	(0.035)	(0.028)	(0.026)	(0.030)
doteal			0.037**					0.044***		
			(0.015)					(0.015)		
absorp					0.051***					0.048***
					(0.014)					(0.015)
comp						0.560**	0.284	0.563**	0.623*	0.520*
						(0.265)	(0.378)	(0.253)	(0.358)	(0.268)
ovrd*comp						-0.074***	0.036	-0.100***	-0.050***	-0.056***
						(0.021)	(0.029)	(0.027)	(0.019)	(0.022)
doteal*comp								0.040***		
								(0.014)		
absorp*comp										-0.012*
										(0.007)
size	0.681***	-0.100**	0.680***	-0.060**	0.697***	0.691***	-0.101**	0.690***	-0.064**	0.704***
	(0.038)	(0.040)	(0.037)	(0.029)	(0.039)	(0.038)	(0.040)	(0.035)	(0.029)	(0.038)
age	0.013*	-0.013*	0.015**	0.006	0.011	0.013*	-0.014*	0.015**	0.007	0.012*
	(0.007)	(0.007)	(0.007)	(0.005)	(0.007)	(0.007)	(0.007)	(0.007)	(0.005)	(0.007)
exper	0.087***	0.076***	0.080***	0.029***	0.084***	0.084***	0.076***	0.069***	0.028***	0.082***
	(0.010)	(0.012)	(0.010)	(0.009)	(0.010)	(0.010)	(0.012)	(0.009)	(0.009)	(0.009)
roe	0.149***	0.130***	0.140***	-0.017	0.158***	0.143***	0.130***	0.134***	-0.018	0.157***
	(0.051)	(0.038)	(0.051)	(0.029)	(0.051)	(0.051)	(0.038)	(0.049)	(0.029)	(0.050)
tobinq	-0.026	0.006	-0.029	0.050***	-0.046*	-0.026	0.006	-0.029	0.051***	-0.042
	(0.029)	(0.015)	(0.031)	(0.009)	(0.028)	(0.029)	(0.015)	(0.029)	(0.009)	(0.028)
cash	-0.033	0.012	-0.029	-0.003	-0.035	-0.030	0.010	-0.022	-0.004	-0.031
	(0.031)	(0.029)	(0.029)	(0.027)	(0.032)	(0.031)	(0.029)	(0.028)	(0.026)	(0.031)
revenue	-0.101*	-0.023	-0.100*	-0.093**	-0.072	-0.101*	-0.023	-0.111**	-0.095**	-0.082
	(0.053)	(0.046)	(0.052)	(0.040)	(0.052)	(0.052)	(0.046)	(0.052)	(0.039)	(0.052)
market	-0.043	-0.111	-0.025	0.086	-0.060	-0.081	-0.108	-0.048	0.075	-0.084
	(0.102)	(0.110)	(0.100)	(0.061)	(0.101)	(0.101)	(0.110)	(0.098)	(0.060)	(0.099)
Constant	-9.303***	4.223***	-9.641***	1.909**	-9.607***	-9.535***	3.824***	-9.941***	1.968**	-9.966***
	(1.098)	(1.310)	(1.091)	(0.792)	(1.103)	(1.085)	(1.319)	(1.037)	(0.793)	(1.096)
Observations	1,110	1,104	1,110	1,110	1,110	1,110	1,104	1,110	1,110	1,110
Pseudo R2	0.81	0.17	0.813	0.199	0.814	0.814	0.171	0.818	0.173	0.818
Wald chi2	1277	99.43	1304	104.3	1322	1325	103.5	1421	120.3	1351
Area FE	YES	YES	YES	YES	YES	YES	YES	YES	YES	YES
Industry FE	YES	YES	YES	YES	YES	YES	YES	YES	YES	YES
Year FE	YES	YES	YES	YES	YES	YES	YES	YES	YES	YES

*** p<0.01,

** p<0.05,

* p<0.1

## 6. Robustness test

Since the dependent variable satisfies the normal distribution after taking the logarithm, we retest each model with the OLS. As shown in Table 6, all hypotheses are verified again through the tests of models 1–11. Model 1 is the test of control variables. Model 2 tests the promotion effect of R&D internationalization on the innovation of the parent company. Model 3 tests the promoting effect of domestic technology alliances on parent company’s innovation. Model 4 is a test of the innovation of the parent company by integrating R&D internationalization and domestic technology alliances. Model 2 confirms Hypothesis 1 and models 2–4 confirm Hypothesis 2. R&D internationalization is confirmed by the mediating effect of domestic technology alliances on innovation of the parent company. Similarly, models 5 and 6 are tests of the mediating effect of absorption capacity, and Hypothesis 3 is confirmed. Models 7–9 and 10–11 are tests of the moderating effect of market competition on domestic technology alliances and absorptive capacity. The moderated mediation model is established, and Hypothesis 4 and Hypothesis 5 are confirmed. At the same time, we change the measurement method of independent variables and use the number of the parent company’s R&D internationalization subsidiaries (ovnrd) as the proxy variable of R&D internationalization. The results are shown in Table 7, and the test results of each model remain consistent with the benchmark model.

**Table 6:** Robustness test results based on linear regression.

VARIABLES	m1	m2	m3	m4	m5	m6	m7	m8	m9	m10	m11
	lnpatent	lnpatent	doteal	lnpatent	absorp	lnpatent	lnpatent	doteal	lnpatent	absorp	lnpatent
lnovrd		0.596***	2.419***	0.505***	1.079**	0.528***	0.578***	2.384***	0.518***	1.058**	0.503***
		(0.171)	(0.468)	(0.181)	(0.508)	(0.166)	(0.170)	(0.469)	(0.179)	(0.494)	(0.168)
doteal				0.037*					0.036*		
				(0.020)					(0.019)		
absorp						0.062***					0.067***
						(0.023)					(0.016)
comp							-0.193	0.876	-0.167	4.916	-0.468
							(0.745)	(2.444)	(0.746)	(3.517)	(0.764)
ovrd*comp							-0.986**	-1.747	-1.239**	-0.099	-0.791*
							(0.484)	(1.064)	(0.506)	(1.882)	(0.476)
doteal*comp									0.107**		
									(0.052)		
absorp*comp											-0.059*
											(0.032)
size	0.760***	0.766***	-0.197	0.773***	-0.127	0.773***	0.770***	-0.191	0.774***	-0.133	0.779***
	(0.069)	(0.066)	(0.139)	(0.066)	(0.167)	(0.066)	(0.066)	(0.139)	(0.065)	(0.167)	(0.067)
age	0.037***	0.036***	-0.045	0.037***	0.018	0.035***	0.037***	-0.044	0.037***	0.018	0.037***
	(0.013)	(0.013)	(0.028)	(0.012)	(0.036)	(0.012)	(0.013)	(0.028)	(0.012)	(0.036)	(0.012)
exper	0.019	0.024	0.046	0.023	0.002	0.024	0.023	0.043	0.023	0.001	0.024
	(0.019)	(0.018)	(0.037)	(0.018)	(0.045)	(0.017)	(0.018)	(0.037)	(0.018)	(0.045)	(0.017)
roe	0.113**	0.099**	0.310***	0.088*	-0.147	0.108**	0.101**	0.312***	0.091**	-0.147	0.117**
	(0.046)	(0.045)	(0.100)	(0.045)	(0.185)	(0.045)	(0.045)	(0.101)	(0.044)	(0.185)	(0.047)
tobinq	-0.006	-0.004	0.030	-0.005	0.302***	-0.023	-0.003	0.031	-0.006	0.302***	-0.020
	(0.017)	(0.017)	(0.052)	(0.018)	(0.100)	(0.018)	(0.017)	(0.052)	(0.017)	(0.099)	(0.017)
cash	0.042	0.041	0.057	0.039	-0.013	0.042	0.041	0.056	0.040	-0.015	0.046
	(0.043)	(0.043)	(0.094)	(0.043)	(0.163)	(0.042)	(0.043)	(0.093)	(0.042)	(0.162)	(0.042)
revenue	-0.094	-0.095	-0.127	-0.090	-0.416*	-0.069	-0.092	-0.124	-0.098	-0.420*	-0.073
	(0.077)	(0.076)	(0.153)	(0.075)	(0.246)	(0.072)	(0.076)	(0.153)	(0.075)	(0.248)	(0.072)
market	-0.161*	-0.161*	-0.267	-0.151	0.265	-0.177*	-0.170*	-0.283	-0.167*	0.265	-0.191**
	(0.094)	(0.095)	(0.302)	(0.095)	(0.326)	(0.093)	(0.095)	(0.302)	(0.095)	(0.328)	(0.093)
Constant	-13.036***	-13.297***	7.725*	-13.585***	3.209	-13.496***	-13.187***	8.291*	-13.270***	3.632	-13.543***
	(1.895)	(1.847)	(4.405)	(1.833)	(4.713)	(1.806)	(1.831)	(4.368)	(1.816)	(4.721)	(1.794)
Observations	1,110	1,110	1,110	1,110	1,110	1,110	1,110	1,110	1,110	1,110	1,110
R-squared	0.638	0.646	0.294	0.649	0.402	0.656	0.648	0.297	0.653	0.404	0.661
F	24.43	24.07	4.697	21.85	3.612	23.40	19.96	4.344	17.32	3.312	19.28
Area FE	YES	YES	YES	YES	YES	YES	YES	YES	YES	YES	YES
Industry FE	YES	YES	YES	YES	YES	YES	YES	YES	YES	YES	YES
Year FE	YES	YES	YES	YES	YES	YES	YES	YES	YES	YES	YES

*** p<0.01,

** p<0.05,

* p<0.1

**Table 7:** Robustness test results based on R&D internationalization variable substitution.

VARIABLES	m1	m2	m3	m4	m5	m6	m7	m8	m9	m10	m11
	patent	patent	doteal	patent	absorp	patent	patent	doteal	patent	absorp	patent
ovnrd		0.114***	0.154***	0.098***	0.062***	0.110***	0.089***	0.156***	0.073***	0.063***	0.085***
		(0.013)	(0.018)	(0.014)	(0.014)	(0.013)	(0.014)	(0.019)	(0.014)	(0.017)	(0.014)
doteal				0.040***					0.040***		
				(0.015)					(0.015)		
absorp						0.055***					0.052***
						(0.014)					(0.014)
comp							0.566**	0.299	0.593**	0.650*	0.517**
							(0.255)	(0.379)	(0.248)	(0.355)	(0.258)
c.c_ovnrd#c.comp							-0.078***	0.011	-0.093***	0.002	-0.079***
							(0.021)	(0.018)	(0.022)	(0.020)	(0.020)
doteal*comp									0.047***		
									(0.013)		
absorp*comp											-0.018***
											(0.007)
size	0.675***	0.662***	-0.109***	0.666***	-0.061**	0.679***	0.676***	-0.113***	0.673***	-0.065**	0.698***
	(0.044)	(0.036)	(0.039)	(0.036)	(0.029)	(0.036)	(0.036)	(0.039)	(0.035)	(0.029)	(0.036)
age	0.017**	0.017**	-0.011	0.018**	0.007	0.014**	0.014*	-0.012	0.015**	0.007	0.013*
	(0.008)	(0.007)	(0.007)	(0.007)	(0.005)	(0.007)	(0.007)	(0.007)	(0.007)	(0.005)	(0.007)
exper	0.033***	0.020***	-0.023**	0.022***	-0.013*	0.020***	0.013*	-0.023**	0.016**	-0.013*	0.013**
	(0.009)	(0.006)	(0.011)	(0.007)	(0.008)	(0.007)	(0.007)	(0.011)	(0.007)	(0.008)	(0.007)
roe	0.185***	0.141***	0.129***	0.130**	-0.018	0.152***	0.157***	0.130***	0.153***	-0.018	0.173***
	(0.055)	(0.050)	(0.038)	(0.051)	(0.029)	(0.050)	(0.054)	(0.038)	(0.054)	(0.029)	(0.053)
tobinq	-0.032	-0.028	0.003	-0.030	0.049***	-0.049*	-0.028	0.003	-0.029	0.049***	-0.044
	(0.033)	(0.029)	(0.015)	(0.030)	(0.009)	(0.027)	(0.030)	(0.015)	(0.030)	(0.009)	(0.028)
cash	-0.028	-0.038	0.011	-0.034	-0.002	-0.040	-0.035	0.011	-0.030	-0.003	-0.035
	(0.035)	(0.030)	(0.029)	(0.028)	(0.027)	(0.031)	(0.029)	(0.029)	(0.028)	(0.026)	(0.030)
revenue	-0.133**	-0.110**	-0.034	-0.107**	-0.098**	-0.080	-0.107**	-0.033	-0.108**	-0.099**	-0.086*
	(0.053)	(0.052)	(0.046)	(0.052)	(0.040)	(0.052)	(0.052)	(0.046)	(0.052)	(0.040)	(0.052)
market	-0.117	-0.023	-0.118	-0.009	0.082	-0.041	-0.031	-0.125	-0.013	0.081	-0.045
	(0.110)	(0.102)	(0.111)	(0.100)	(0.061)	(0.101)	(0.100)	(0.112)	(0.097)	(0.061)	(0.098)
Constant	-8.780***	-9.220***	4.243***	-9.597***	1.833**	-9.527***	-9.256***	4.590***	-9.451***	2.111**	-9.848***
	(1.133)	(1.073)	(1.301)	(1.069)	(0.794)	(1.076)	(1.047)	(1.316)	(1.029)	(0.820)	(1.063)
Observations	1,110	1,110	1,104	1,110	1,110	1,110	1,110	1,104	1,110	1,110	1,110
Pseudo R2	0.7939	0.8125	0.1674	0.8146	0.1976	0.8168	0.8176	0.1678	0.8210	0.1994	0.8225
Wald chi2	979.9	1296	97.70	1327	99.41	1329	1304	98.12	1359	104.3	1342
Area FE	YES	YES	YES	YES	YES	YES	YES	YES	YES	YES	YES
Industry FE	YES	YES	YES	YES	YES	YES	YES	YES	YES	YES	YES
Year FE	YES	YES	YES	YES	YES	YES	YES	YES	YES	YES	YES

*** p<0.01,

** p<0.05,

* p<0.1

## 7. Conclusions and discussion

### 7.1. Conclusions and contributions

According to the traditional international investment theory, the most important reason for enterprises to make foreign investment is "internalization", that is, competition barriers and high transaction costs lead to the incomplete structural market, and meanwhile, the knowledge market is not complete due to the difficulty in obtaining the relevant knowledge information of production and sales, therefore, when the market transaction cost of an enterprise is far greater than the cost of "internalization", the enterprise is more willing to fix the intermediate products such as knowledge and technology inside the enterprise [46, 47], the overseas investment thus made is a kind of "asset-exploiting investment", which is based on the ability of multinational enterprises not only to prevent the loss of core technology assets, but also to obtain a broader market and sales and supply network [35]. However, cross-border investment of emerging market enterprises is totally different. Many emerging market enterprises are faced with domestic institutional difficulties and weak economic environment, and they do not have absolute advantages such as technology, brand and sales network. Therefore, these enterprises hope to obtain core competitive resources through rapid internationalization [1,48]. R&D internationalization is the most direct and rapid way to acquire technological resources. Existing literature focuses on the motivation and expansion methods of internationalization of emerging market firms [49, 50], lack of mechanism research on the internationalization of emerging market enterprise, which is still a gap in the research in the emerging market enterprise and their overseas R&D activities. Studying the mechanism of the R&D internationalization of emerging market enterprise is of great significance for us to understand the catch-up path of emerging market firms and to supplement and expand the existing international business theories. At the same time, the research on the mechanism of R&D internationalization also helps managers to pay attention to the factors that multinational enterprises may ignore in the process of internationalization: the absorptive capacity of enterprise and local stakeholders, so as to better promote enterprise innovation and performance.

Can R&D internationalization promote the innovation of late-development enterprises? The knowledge-based view emphasizes that the knowledge accumulated by enterprises is the key to the sustainable innovation ability of enterprises, and also the key to the core competitiveness of enterprises [55]. Innovation scholars believe that novel innovations are often the result of existing and diversified knowledge combinations [28], and that R&D internationalization creates the basis for the cultivation of diverse knowledge within an enterprise. In this paper, we found that China’s R&D internationalization played a significant role in promoting the innovation of the parent company, and we illustrated for the first time, R&D internationalization, domestic technology alliances and absorptive capacity were combined in a unified research framework, which proved the role of domestic technology alliances and absorptive capacity in the connection between R&D internationalization and innovation progress, market competition played a moderating role in this process. In fierce market competition, domestic technology alliances played a positively significant role in promoting innovation while the absorptive capability of parent company played a negative one. The theoretical and empirical contributions of this paper are as follows:

First, our study complements existing international investment theories and is a useful complement to the existing literature on the internationalization of emerging market firms. Based on the theoretical basis of Hymer, Buckley and Casson [46, 145], Dunning integrated ownership advantage, internalization advantage theory and location advantage theory [34], and believed that the ownership advantage of enterprises on specific assets was the basis of enterprises’ cross-border investment. Such advantages include the advantages inherent in the enterprise itself, such as technology, trademark, management skills, also include the advantages generated through foreign investment, such as diversification of products and markets, integration of production processes, and monopoly of sales markets and raw materials. The purpose of internationalization of multinational corporations is to use their assets and ownership for internal use. They choose the regions with specific geographical advantages to invest in order to obtain the maximum benefits. However, the traditional international business theory is mainly aimed at the mature market countries, and ignores the technology seeking motivation of enterprises to a certain extent. For emerging market countries, their internationalization is mainly aimed at technology acquisition and learning effect [146, 147]. The existing research on emerging market internationalization focuses on the motivation and entry mode of emerging market enterprises’ internationalization [139, 147]. Our research on the impact of R&D internationalization on innovation of emerging market firms confirms the technology seeking motivation of emerging market firms. And on the basis of the previous literature, this paper expounds the path of R&D internationalization of emerging market firms, which promotes the innovation of emerging market enterprises through the absorptive capacity of local parent companies and the role of domestic technology alliance, while fierce local market competition promotes this role. These mechanisms have not been identified in previous studies. This finding is crucial for us to expand existing research on emerging multinational firm internationalization and better understand how these firms achieve innovation and thus improve firm performance.

Second, our findings speak to one of the most essential and timely discussions of the emerging market enterprise internationalization—the international R&D activities. With the development of emerging market enterprises such as China and India, the world investment pattern is undergoing great changes. How do these enterprises achieve a leap in their own performance through international investment as a springboard? This problem has received more and more academic attention. Take China as an example, in recent years, China’s Huawei has set up dozens of international R&D centers in France, Japan, India, Germany, Indonesia and other places, and has made great achievements in communication software, cutting-edge communication network services, chip manufacturing and other areas. Bytedance, a new Chinese tech company that has risen rapidly in recent years with its artificial intelligence algorithms, has greatly expanded its overseas operations, setting up research labs in the United States, Japan, Europe and other regions. Our research has focused on the international R&D activities, explains the path and the mechanism of R&D internationalization in emerging markets, not only help us to expand international business theory, but also help us to understand the phenomenon of the rise of international R&D activities in emerging market, confirmed the importance of these activities. And for the first time, we analyze the relationship between R&D internationalization, domestic technology alliance and absorptive capacity of parent company deeply, which is of great significance to explain the phenomenon of overseas R&D activities in emerging markets.

Third, similarly, academia is also abuzz on the subject of the effect of changing environment inside or outside the MNE (muti-national enterprise). Complementing previous studies, our paper extends the MNE literature and sheds new light on the understudied aspects of effect of domestic environment on promoting the RD internationalization. Domestic technology alliances play a crucial role in promoting innovation of emerging market firms’ R&D internationalization. Meanwhile, the intensity of local market competition significantly moderates the effect of local technology alliances on the firm’s innovation performance. While previous studies have focused on the motivation of knowledge acquisition in emerging market enterprises and studied overseas R&D activities and local R&D activities separately, our research focuses on the important local factors including absorptive capacity of the parent company and domestic technology alliance, perceives them as a whole to promote knowledge integration after the acquisition of knowledge in developed markets and sees them are the key factors to innovation catch-up. Ignoring these factors may lead to a patchwork and superficial understanding of innovation catch-up in emerging markets. Moreover, previous literatures focus on the influencing factors at the host country level and the defects of the home country system in the process of the internationalization of emerging market enterprises [1, 148], ignoring the dual effect of competitive environment to local R&D activities may lead to managers’ unbalanced allocation of resources and inability to adjust the enterprise’s strategic decisions in time.

Fourth, starting from the perspective of knowledge-based view, we used a unified framework to link the R&D internationalization of emerging market enterprises with local technology alliances, which not only expands the research related to international investment, but also enriches the relevant content of knowledge-based view. Knowledge-based view holds that knowledge which is implicit and unteachable is the root of enterprise heterogeneity and deeply embedded in the local environment [52, 55]. But it is mainly focus on the integration and acquisition of internal knowledge, and we believe that the integration and understanding of knowledge by technology alliances in external environments is critical. The understanding and digestion of knowledge by multi-stakeholder groups can make better use of knowledge and feedback knowledge results. Such interest groups not only include upstream and downstream enterprises of the supply chain, but also non-profit organizations such as research institutes and universities. External synergy is beneficial to enhance the ability of the core partner in the alliance to use knowledge, this expands our understanding of knowledge-based theory and absorptive capacity theory.

### 7.2. Implications

The conclusions of this paper have the following policy implications for emerging market enterprises to deepen the reform of scientific and technological systems and promote the technological innovation of enterprises. From the perspective of the government, the government should first pay much attention to the promotion effect of R&D internationalization on technological progress. Due to the increasingly fierce competition between developed and developing countries in the overseas market in recent years, it is not easy for the government to acquire technologies through R&D internationalization, so it is necessary for the government to realize the important strategic position and the practical difficulties faced in R&D internationalization. Second, the government should attach importance to domestic technology alliances for fostering independent innovation; the government should provide more financial support and policy guidance to enterprises with international R&D business and domestic technology alliance business, and continue to improve laws and regulations on cooperation forms such as "industry-university-research cooperation", "public innovation and sharing", and "enterprise intermediary consulting service platforms", promote the communication and connection among stakeholders such as universities, research institutes, industry associations, suppliers and enterprise competitors, build good cooperation platforms for domestic technology alliances, and make domestic technology alliances an important base for the transformation of R&D internationalization achievements. Thirdly, the government should pay much attention to the cultivation of the absorptive capacity of domestic enterprises through talent cultivation support, innovative hardware facilities construction, and financial market stability to provide a sound environment for the cultivation of absorptive capacity. From the perspective of enterprises, first of all, emerging market enterprises should not only aim at expanding the market or reducing the production cost of products, but also pay more attention to the role of knowledge assets. Secondly, It is not only for enterprises to "go out" and get close to the innovation frontier to acquire the core knowledge assets of the host country, but in addition, it is also necessary for enterprises to attract technical talents by means of improving salary and treatment, so as to enhance the absorption capacity of enterprises. Finally, enterprises need to consciously cooperate with domestic stakeholders and establish a win-win relationship with suppliers, universities, customers and even competitors in order to stabilize their strategic position in the face of fierce market competition.

### 7.3. Limitations and future research directions

This paper has the following deficiencies: First, we only use the panel data of a single country, China, while it is worth further exploring in the future whether the R&D internationalization performance of India, Brazil and other late-developing countries in economic and social transformation can promote the innovation of parent companies through similar paths. Second, restricted by data availability, in this paper, we only apply the samples from 2012 to 2017 as the research object, meanwhile, we select the number of R&D internationalization subsidiaries accounting for the number of overseas investment subsidiaries as the proxy variable of R&D internationalization, such proxy variables are more specific and accurate than binary dummy variables commonly used at present, however, it still has limitations and remains to be further perfected. Third, this paper only focuses on the relationship between domestic technology alliances, absorptive capacity and R&D internationalization, and the domestic technology alliances, absorptive capacity of R&D internationalization on innovation of the parent company play a partial mediating role, however, as for the existence of other mediators, the mechanism of action and the path of action of other mediators, this study has not been carried out yet. Future research can further explore the mediating effect between R&D internationalization and the innovation performance of the parent company.

## Supporting information

S1 Fig Logic frame figure. (TIF)

S1 Table Variable source and definition table. (DOCX)

S2 Table Variable relationship description table. (DOCX)

S3 Table Mediating effect test and moderated mediating effect test. (DOCX)

S4 Table Regression results of the first stage. (DOCX)

S5 Table Regression results of 2SLS. (DOCX)

S6 Table Robustness test results based on linear regression. (DOCX)

S7 Table Robustness test results based on R&D internationalization variable substitution. (DOCX)

S1 Data (RAR)
