# Supplementary figures and images for: RD internationalization, domestic technology alliance, and innovation in emerging market
Source: PLoS One. 2021 Jun 25;16(6):e0252669. doi: 10.1371/journal.pone.0252669 (PMC8232540; doi:10.1371/journal.pone.0252669)

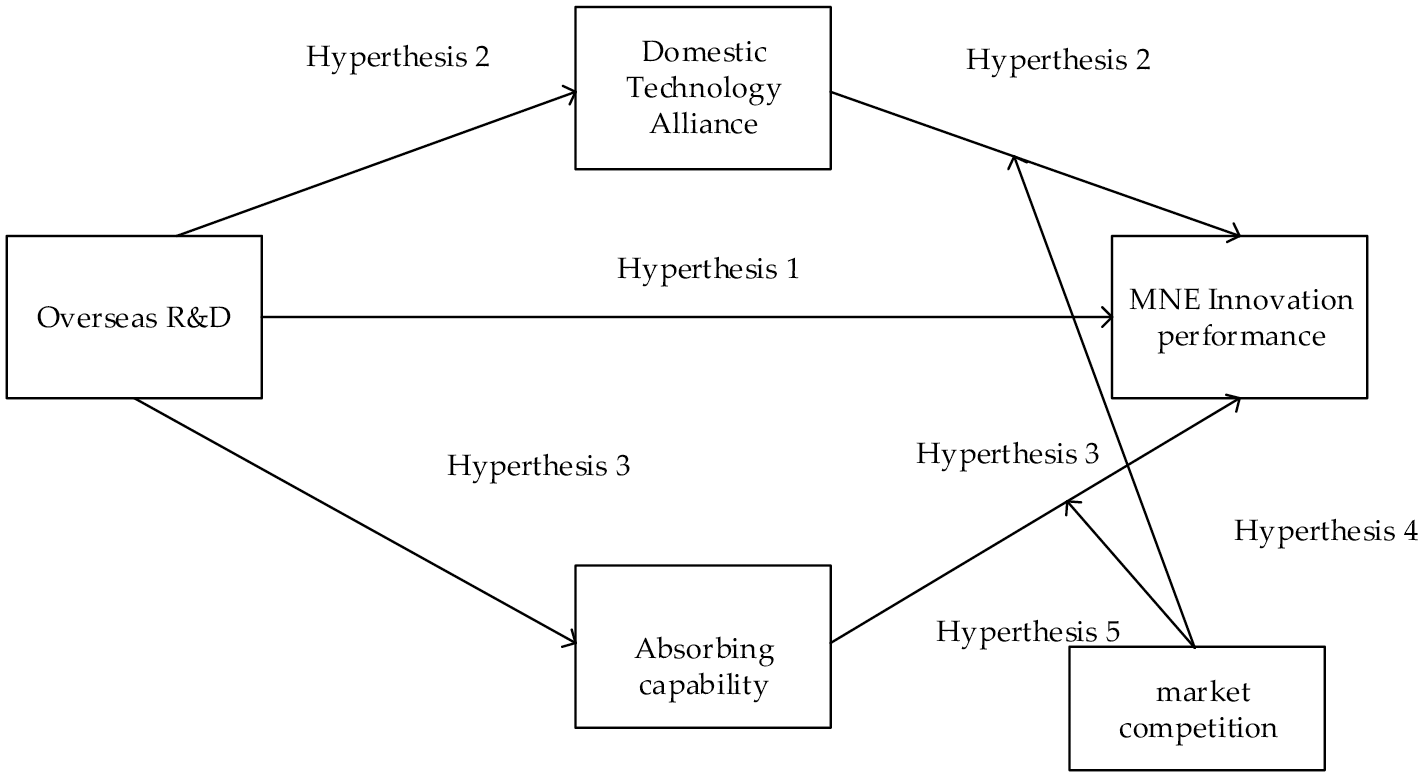

Supplement: S1 Fig — (TIF) [file pone.0252669.s001.tif]
