# Supplementary material for: RD internationalization, domestic technology alliance, and innovation in emerging market
Source: PLoS One. 2021 Jun 25;16(6):e0252669. doi: 10.1371/journal.pone.0252669 (PMC8232540; doi:10.1371/journal.pone.0252669)
Supplement: S1 Table — (DOCX) [file pone.0252669.s002.docx]

**S1 Table.** Variable source and definition table

| Variable types | symbol | description | observations | mean | sd | minimum | maximum |
| --- | --- | --- | --- | --- | --- | --- | --- |
| Explained variable | patent | Innovation performance | 1,110 | 190.454 | 404.659 | 2.000 | 2616.000 |
| explanatory variable | ovrd | R&D internationalization | 1,110 | 0.323 | 0.380 | 0.000 | 1.000 |
| mediating variables | doteal | Domestic Technology Alliance | 1,110 | 2.310 | 2.603 | 0.000 | 10.000 |
|  | absorp | Absorptive capacity | 1,110 | 3.751 | 3.218 | 0.306 | 21.439 |
| moderating variable | comp | Market competition | 1,110 | 0.977 | 0.350 | 0.474 | 1.886 |
| Control variables | size | Enterprise size (logarithm) | 1,110 | 22.287 | 1.182 | 20.143 | 25.715 |
|  | age | Enterprise age | 1,110 | 14.978 | 5.373 | 6.000 | 37.000 |
|  | exper | Overseas Investment experience | 1,110 | 3.929 | 3.482 | 1.000 | 21.000 |
|  | roe | Return on equity | 1,110 | 0.081 | 0.138 | -2.179 | 1.611 |
|  | tobinq | The market value of the business divided by the replacement cost of the assets | 1,110 | 2.625 | 2.518 | 0.113 | 33.475 |
|  | cash | Cash flow (logarithm) | 1,110 | 20.368 | 1.219 | 17.536 | 24.107 |
|  | revenue | The intensity of tax | 1,110 | 0.015 | 0.019 | -0.207 | 0.145 |
|  | market | Institutional quality | 1,110 | 0.708 | 0.110 | 0.284 | 0.836 |
| Instrumental variable | alls | Overseas investment scale of companies in other industries other than the parent company (logarithm) | 1,110 | 6.489 | 0.253 | 5.852 | 6.958 |
