# Supplementary material for: RD internationalization, domestic technology alliance, and innovation in emerging market
Source: PLoS One. 2021 Jun 25;16(6):e0252669. doi: 10.1371/journal.pone.0252669 (PMC8232540; doi:10.1371/journal.pone.0252669)
Supplement: S2 Table — (DOCX) [file pone.0252669.s003.docx]

**S2 Table.** Variable relationship description table

|  | **patent** | **ovrd** | **doteal** | **absorp** | **comp** | **size** | **Age** | **exper** | **roe** | **tobinq** | **cash** | **revenue** | **market** |
| --- | --- | --- | --- | --- | --- | --- | --- | --- | --- | --- | --- | --- | --- |
| patent | 1.0000 |  |  |  |  |  |  |  |  |  |  |  |  |
| ovrd | 0.126*** | 1.0000 |  |  |  |  |  |  |  |  |  |  |  |
| doteal | 0.023 | 0.090*** | 0.111*** |  |  |  |  |  |  |  |  |  |  |
| absorp | 0.001 | -0.069** | -0.084*** | 1.0000 |  |  |  |  |  |  |  |  |  |
| comp | -0.045 | -0.042 | -0.080*** | -0.144*** | 1.0000 |  |  |  |  |  |  |  |  |
| size | 0.564*** | -0.008 | -0.016 | -0.132*** | -0.148*** | 1.0000 |  |  |  |  |  |  |  |
| age | 0.094*** | -0.166*** | 0.047 | -0.055** | -0.064** | 0.239*** | 1.0000 |  |  |  |  |  |  |
| exper | 0.299*** | 0.081*** | 0.124*** | -0.021 | -0.028 | 0.430*** | 0.125*** | 1.0000 |  |  |  |  |  |
| roe | 0.111*** | 0.027 | 0.109*** | 0.011 | -0.007 | 0.114*** | 0.011 | 0.032 | 1.0000 |  |  |  |  |
| tobinq | -0.204*** | 0.020 | -0.012 | 0.275*** | 0.275*** | -0.381*** | -0.111*** | -0.118*** | 0.205*** | 1.0000 |  |  |  |
| cash | 0.148*** | 0.012 | -0.010 | 0.006 | 0.009 | 0.279*** | 0.197*** | 0.139*** | 0.006 | -0.091*** | 1.0000 |  |  |
| revenue | -0.117*** | -0.042 | -0.005 | 0.018 | 0.007 | -0.034 | -0.029 | -0.070** | 0.143*** | 0.144*** | 0.001 | 1.0000 |  |
| market | -0.052* | 0.020 | -0.012 | 0.135*** | 0.135*** | -0.011 | -0.039 | 0.056* | 0.033 | 0.102*** | 0.113*** | 0.03 | 1.0000 |
