# Supplementary material for: RD internationalization, domestic technology alliance, and innovation in emerging market
Source: PLoS One. 2021 Jun 25;16(6):e0252669. doi: 10.1371/journal.pone.0252669 (PMC8232540; doi:10.1371/journal.pone.0252669)
Supplement: S3 Table — (DOCX) [file pone.0252669.s004.docx]

**S3 Table.** Mediating effect test and moderated mediating effect test

|  | **m1** | **m2** | **m3** | **m4** | **m5** | **m6** | **m7** | **m8** | **m9** | **m10** | **m11** |
| --- | --- | --- | --- | --- | --- | --- | --- | --- | --- | --- | --- |
| VARIABLES | patent | patent | doteal | patent | absorp | patent | patent | doteal | patent | absorp | patent |
| ovrd |  | 0.479*** | 0.707*** | 0.349*** | 0.160** | 0.441*** | 0.250*** | 0.360*** | 0.217*** | 0.095** | 0.231*** |
|  |  | (0.107) | (0.092) | (0.106) | (0.065) | (0.110) | (0.039) | (0.069) | (0.040) | (0.046) | (0.038) |
| doteal |  |  |  | 0.058*** |  |  |  |  | 0.059*** |  |  |
|  |  |  |  | (0.015) |  |  |  |  | (0.015) |  |  |
| absorp |  |  |  |  |  | 0.056*** |  |  |  |  | 0.053*** |
|  |  |  |  |  |  | (0.015) |  |  |  |  | (0.015) |
| comp |  |  |  |  |  |  | 0.498** | 0.212 | 0.535** | 0.641* | 0.452* |
|  |  |  |  |  |  |  | (0.247) | (0.366) | (0.241) | (0.361) | (0.251) |
| ovrd*comp |  |  |  |  |  |  | -0.401*** | -0.255** | -0.408*** | 0.006 | -0.340*** |
|  |  |  |  |  |  |  | (0.098) | (0.100) | (0.106) | (0.073) | (0.095) |
| doteal*comp |  |  |  |  |  |  |  |  | 0.039*** |  |  |
|  |  |  |  |  |  |  |  |  | (0.014) |  |  |
| absorp*comp |  |  |  |  |  |  |  |  |  |  | -0.012* |
|  |  |  |  |  |  |  |  |  |  |  | (0.007) |
| size | 0.675*** | 0.680*** | -0.074** | 0.683*** | -0.052* | 0.695*** | 0.667*** | -0.108*** | 0.669*** | -0.062** | 0.683*** |
|  | (0.044) | (0.040) | (0.038) | (0.038) | (0.030) | (0.040) | (0.040) | (0.039) | (0.038) | (0.030) | (0.040) |
| age | 0.017** | 0.015** | -0.014* | 0.018** | 0.005 | 0.013* | 0.018** | -0.011 | 0.020** | 0.005 | 0.017** |
|  | (0.008) | (0.008) | (0.007) | (0.008) | (0.005) | (0.008) | (0.008) | (0.007) | (0.008) | (0.005) | (0.008) |
| exper | 0.033*** | 0.041*** | 0.021** | 0.040*** | 0.000 | 0.040*** | 0.044*** | 0.021** | 0.044*** | -0.000 | 0.044*** |
|  | (0.009) | (0.008) | (0.010) | (0.007) | (0.007) | (0.008) | (0.008) | (0.010) | (0.008) | (0.007) | (0.008) |
| roe | 0.185*** | 0.162*** | 0.109*** | 0.143*** | -0.016 | 0.175*** | 0.160*** | 0.126*** | 0.143*** | -0.014 | 0.177*** |
|  | (0.055) | (0.053) | (0.036) | (0.053) | (0.029) | (0.053) | (0.052) | (0.037) | (0.051) | (0.030) | (0.051) |
| tobinq | -0.032 | -0.031 | 0.007 | -0.034 | 0.050*** | -0.050* | -0.032 | 0.005 | -0.034 | 0.049*** | -0.048* |
|  | (0.033) | (0.031) | (0.014) | (0.032) | (0.009) | (0.029) | (0.031) | (0.015) | (0.031) | (0.009) | (0.029) |
| cash | -0.028 | -0.032 | 0.016 | -0.028 | 0.003 | -0.033 | -0.040 | 0.005 | -0.033 | 0.001 | -0.040 |
|  | (0.035) | (0.032) | (0.029) | (0.030) | (0.027) | (0.034) | (0.033) | (0.029) | (0.030) | (0.026) | (0.034) |
| revenue | -0.133** | -0.130** | -0.034 | -0.122** | -0.098** | -0.103** | -0.126** | -0.030 | -0.122** | -0.098** | -0.106** |
|  | (0.053) | (0.052) | (0.043) | (0.052) | (0.040) | (0.052) | (0.051) | (0.043) | (0.052) | (0.040) | (0.052) |
| market | -0.117 | -0.082 | -0.119 | -0.056 | 0.079 | -0.101 | -0.109 | -0.116 | -0.080 | 0.079 | -0.120 |
|  | (0.110) | (0.104) | (0.108) | (0.101) | (0.062) | (0.104) | (0.104) | (0.110) | (0.100) | (0.062) | (0.103) |
| Constant | -8.780*** | -9.285*** | 3.169** | -9.800*** | 1.547** | -9.568*** | -8.450*** | 4.324*** | -8.904*** | 1.949** | -8.918*** |
|  | (1.133) | (1.114) | (1.276) | (1.094) | (0.786) | (1.118) | (1.086) | (1.286) | (1.060) | (0.835) | (1.093) |
| Observations | 1,110 | 1,110 | 1,104 | 1,110 | 1,110 | 1,110 | 1,110 | 1,104 | 1,110 | 1,110 | 1,110 |
| Pseudo R2 | 0.794 | 0.802 | 0.174 | 0.807 | 0.196 | 0.806 | 0.806 | 0.166 | 0.811 | 0.196 | 0.81 |
| Waldchi2 | 979.9 | 1114 | 101.3 | 1191 | 90.88 | 1136 | 1169 | 74.37 | 1301 | 90.63 | 1174 |
| Area FE | YES | YES | YES | YES | YES | YES | YES | YES | YES | YES | YES |
| Industry FE | YES | YES | YES | YES | YES | YES | YES | YES | YES | YES | YES |
| Year FE | YES | YES | YES | YES | YES | YES | YES | YES | YES | YES | YES |
| VIF | 1.21 | 1.20 | 1.20 | 1.20 | 1.20 | 1.20 | 1.18 | 1.18 | 1.19 | 1.18 | 1.20 |
