# Supplementary material for: RD internationalization, domestic technology alliance, and innovation in emerging market
Source: PLoS One. 2021 Jun 25;16(6):e0252669. doi: 10.1371/journal.pone.0252669 (PMC8232540; doi:10.1371/journal.pone.0252669)
Supplement: S4 Table — (DOCX) [file pone.0252669.s005.docx]

**S4 Table.** Regression results of the first stage

|  | m1 | m2 | m3 | m4 | m5 | m6 | m7 | m8 | m9 | m10 |
| --- | --- | --- | --- | --- | --- | --- | --- | --- | --- | --- |
| VARIABLES | ovrd | ovrd | ovrd | ovrd | ovrd | ovrd | ovrd | ovrd | ovrd | ovrd |
| alls | -0.073** | -0.073** | -0.071** | -0.073** | -0.070** | -0.076** | -0.076** | -0.075** | -0.076** | -0.073** |
|  | (0.035) | (0.035) | (0.034) | (0.035) | (0.035) | (0.035) | (0.035) | (0.035) | (0.035) | (0.035) |
| RDS | -0.185*** | -0.185*** | -0.180*** | -0.185*** | -0.184*** | -0.183*** | -0.183*** | -0.178*** | -0.183*** | -0.183*** |
|  | (0.006) | (0.006) | (0.006) | (0.006) | (0.006) | (0.006) | (0.006) | (0.006) | (0.006) | (0.006) |
| Constant | 1.269*** | 1.269*** | 1.183*** | 1.269*** | 1.245*** | 1.281*** | 1.281*** | 1.228*** | 1.281*** | 1.264*** |
|  | (0.281) | (0.281) | (0.281) | (0.281) | (0.281) | (0.281) | (0.281) | (0.281) | (0.281) | (0.282) |
| Observations | 1,110 | 1,110 | 1,110 | 1,110 | 1,110 | 1,110 | 1,110 | 1,110 | 1,110 | 1,110 |
| R-squared | 0.499 | 0.499 | 0.503 | 0.499 | 0.500 | 0.501 | 0.501 | 0.506 | 0.501 | 0.502 |
| F | 109.5 | 109.5 | 101.1 | 109.5 | 99.90 | 91.64 | 91.64 | 79.95 | 91.64 | 78.74 |
| Kleibergen-Paaprk | 123.778 | 123.778 | 112.832 | 123.778 | 122.587 | 121.668 | 121.668 | 112.588 | 121.668 | 120.644 |
| Controls | YES | YES | YES | YES | YES | YES | YES | YES | YES | YES |
