# Supplementary material for: RD internationalization, domestic technology alliance, and innovation in emerging market
Source: PLoS One. 2021 Jun 25;16(6):e0252669. doi: 10.1371/journal.pone.0252669 (PMC8232540; doi:10.1371/journal.pone.0252669)
Supplement: S5 Table — (DOCX) [file pone.0252669.s006.docx]

**S5 Table.** Regression results of 2SLS

|  | m1 | m2 | m3 | m4 | m5 | m6 | m7 | m8 | m9 | m10 |
| --- | --- | --- | --- | --- | --- | --- | --- | --- | --- | --- |
| VARIABLES | patent | doteal | patent | absorp | patent | patent | doteal | patent | absorp | patent |
| ovrd | 0.213*** | 0.295*** | 0.185*** | 0.121*** | 0.204*** | 0.211*** | 0.304*** | 0.188*** | 0.106*** | 0.204*** |
|  | (0.029) | (0.035) | (0.031) | (0.025) | (0.030) | (0.029) | (0.035) | (0.028) | (0.026) | (0.030) |
| doteal |  |  | 0.037** |  |  |  |  | 0.044*** |  |  |
|  |  |  | (0.015) |  |  |  |  | (0.015) |  |  |
| absorp |  |  |  |  | 0.051*** |  |  |  |  | 0.048*** |
|  |  |  |  |  | (0.014) |  |  |  |  | (0.015) |
| comp |  |  |  |  |  | 0.560** | 0.284 | 0.563** | 0.623* | 0.520* |
|  |  |  |  |  |  | (0.265) | (0.378) | (0.253) | (0.358) | (0.268) |
| ovrd*comp |  |  |  |  |  | -0.074*** | 0.036 | -0.100*** | -0.050*** | -0.056** |
|  |  |  |  |  |  | (0.021) | (0.029) | (0.027) | (0.019) | (0.022) |
| doteal*comp |  |  |  |  |  |  |  | 0.040*** |  |  |
|  |  |  |  |  |  |  |  | (0.014) |  |  |
| absorp*comp |  |  |  |  |  |  |  |  |  | -0.012* |
|  |  |  |  |  |  |  |  |  |  | (0.007) |
| size | 0.681*** | -0.100** | 0.680*** | -0.060** | 0.697*** | 0.691*** | -0.101** | 0.690*** | -0.064** | 0.704*** |
|  | (0.038) | (0.040) | (0.037) | (0.029) | (0.039) | (0.038) | (0.040) | (0.035) | (0.029) | (0.038) |
| age | 0.013* | -0.013* | 0.015** | 0.006 | 0.011 | 0.013* | -0.014* | 0.015** | 0.007 | 0.012* |
|  | (0.007) | (0.007) | (0.007) | (0.005) | (0.007) | (0.007) | (0.007) | (0.007) | (0.005) | (0.007) |
| exper | 0.087*** | 0.076*** | 0.080*** | 0.029*** | 0.084*** | 0.084*** | 0.076*** | 0.069*** | 0.028*** | 0.082*** |
|  | (0.010) | (0.012) | (0.010) | (0.009) | (0.010) | (0.010) | (0.012) | (0.009) | (0.009) | (0.009) |
| roe | 0.149*** | 0.130*** | 0.140*** | -0.017 | 0.158*** | 0.143*** | 0.130*** | 0.134*** | -0.018 | 0.157*** |
|  | (0.051) | (0.038) | (0.051) | (0.029) | (0.051) | (0.051) | (0.038) | (0.049) | (0.029) | (0.050) |
| tobinq | -0.026 | 0.006 | -0.029 | 0.050*** | -0.046* | -0.026 | 0.006 | -0.029 | 0.051*** | -0.042 |
|  | (0.029) | (0.015) | (0.031) | (0.009) | (0.028) | (0.029) | (0.015) | (0.029) | (0.009) | (0.028) |
| cash | -0.033 | 0.012 | -0.029 | -0.003 | -0.035 | -0.030 | 0.010 | -0.022 | -0.004 | -0.031 |
|  | (0.031) | (0.029) | (0.029) | (0.027) | (0.032) | (0.031) | (0.029) | (0.028) | (0.026) | (0.031) |
| revenue | -0.101* | -0.023 | -0.100* | -0.093** | -0.072 | -0.101* | -0.023 | -0.111** | -0.095** | -0.082 |
|  | (0.053) | (0.046) | (0.052) | (0.040) | (0.052) | (0.052) | (0.046) | (0.052) | (0.039) | (0.052) |
| market | -0.043 | -0.111 | -0.025 | 0.086 | -0.060 | -0.081 | -0.108 | -0.048 | 0.075 | -0.084 |
|  | (0.102) | (0.110) | (0.100) | (0.061) | (0.101) | (0.101) | (0.110) | (0.098) | (0.060) | (0.099) |
| Constant | -9.303*** | 4.223*** | -9.641*** | 1.909** | -9.607*** | -9.535*** | 3.824*** | -9.941*** | 1.968** | -9.966*** |
|  | (1.098) | (1.310) | (1.091) | (0.792) | (1.103) | (1.085) | (1.319) | (1.037) | (0.793) | (1.096) |
| Observations | 1,110 | 1,104 | 1,110 | 1,110 | 1,110 | 1,110 | 1,104 | 1,110 | 1,110 | 1,110 |
| Pseudo R2 | 0.81 | 0.17 | 0.813 | 0.199 | 0.814 | 0.814 | 0.171 | 0.818 | 0.173 | 0.818 |
| Wald chi2 | 1277 | 99.43 | 1304 | 104.3 | 1322 | 1325 | 103.5 | 1421 | 120.3 | 1351 |
| Area FE | YES | YES | YES | YES | YES | YES | YES | YES | YES | YES |
| Industry FE | YES | YES | YES | YES | YES | YES | YES | YES | YES | YES |
| Year FE | YES | YES | YES | YES | YES | YES | YES | YES | YES | YES |
