# Supplementary material for: RD internationalization, domestic technology alliance, and innovation in emerging market
Source: PLoS One. 2021 Jun 25;16(6):e0252669. doi: 10.1371/journal.pone.0252669 (PMC8232540; doi:10.1371/journal.pone.0252669)
Supplement: S6 Table — (DOCX) [file pone.0252669.s007.docx]

**S6 Table.** Robustness test results based on linear regression

|  | m1 | m2 | m3 | m4 | m5 | m6 | m7 | m8 | m9 | m10 | m11 |
| --- | --- | --- | --- | --- | --- | --- | --- | --- | --- | --- | --- |
| VARIABLES | lnpatent | lnpatent | doteal | lnpatent | absorp | lnpatent | lnpatent | doteal | lnpatent | absorp | lnpatent |
| lnovrd |  | 0.596*** | 2.419*** | 0.505*** | 1.079** | 0.528*** | 0.578*** | 2.384*** | 0.518*** | 1.058** | 0.503*** |
|  |  | (0.171) | (0.468) | (0.181) | (0.508) | (0.166) | (0.170) | (0.469) | (0.179) | (0.494) | (0.168) |
| doteal |  |  |  | 0.037* |  |  |  |  | 0.036* |  |  |
|  |  |  |  | (0.020) |  |  |  |  | (0.019) |  |  |
| absorp |  |  |  |  |  | 0.062*** |  |  |  |  | 0.067*** |
|  |  |  |  |  |  | (0.023) |  |  |  |  | (0.016) |
| comp |  |  |  |  |  |  | -0.193 | 0.876 | -0.167 | 4.916 | -0.468 |
|  |  |  |  |  |  |  | (0.745) | (2.444) | (0.746) | (3.517) | (0.764) |
| ovrd*comp |  |  |  |  |  |  | -0.986** | -1.747 | -1.239** | -0.099 | -0.791* |
|  |  |  |  |  |  |  | (0.484) | (1.064) | (0.506) | (1.882) | (0.476) |
| doteal*comp |  |  |  |  |  |  |  |  | 0.107** |  |  |
|  |  |  |  |  |  |  |  |  | (0.052) |  |  |
| absorp*comp |  |  |  |  |  |  |  |  |  |  | -0.059* |
|  |  |  |  |  |  |  |  |  |  |  | (0.032) |
| size | 0.760*** | 0.766*** | -0.197 | 0.773*** | -0.127 | 0.773*** | 0.770*** | -0.191 | 0.774*** | -0.133 | 0.779*** |
|  | (0.069) | (0.066) | (0.139) | (0.066) | (0.167) | (0.066) | (0.066) | (0.139) | (0.065) | (0.167) | (0.067) |
| age | 0.037*** | 0.036*** | -0.045 | 0.037*** | 0.018 | 0.035*** | 0.037*** | -0.044 | 0.037*** | 0.018 | 0.037*** |
|  | (0.013) | (0.013) | (0.028) | (0.012) | (0.036) | (0.012) | (0.013) | (0.028) | (0.012) | (0.036) | (0.012) |
| exper | 0.019 | 0.024 | 0.046 | 0.023 | 0.002 | 0.024 | 0.023 | 0.043 | 0.023 | 0.001 | 0.024 |
|  | (0.019) | (0.018) | (0.037) | (0.018) | (0.045) | (0.017) | (0.018) | (0.037) | (0.018) | (0.045) | (0.017) |
| roe | 0.113** | 0.099** | 0.310*** | 0.088* | -0.147 | 0.108** | 0.101** | 0.312*** | 0.091** | -0.147 | 0.117** |
|  | (0.046) | (0.045) | (0.100) | (0.045) | (0.185) | (0.045) | (0.045) | (0.101) | (0.044) | (0.185) | (0.047) |
| tobinq | -0.006 | -0.004 | 0.030 | -0.005 | 0.302*** | -0.023 | -0.003 | 0.031 | -0.006 | 0.302*** | -0.020 |
|  | (0.017) | (0.017) | (0.052) | (0.018) | (0.100) | (0.018) | (0.017) | (0.052) | (0.017) | (0.099) | (0.017) |
| cash | 0.042 | 0.041 | 0.057 | 0.039 | -0.013 | 0.042 | 0.041 | 0.056 | 0.040 | -0.015 | 0.046 |
|  | (0.043) | (0.043) | (0.094) | (0.043) | (0.163) | (0.042) | (0.043) | (0.093) | (0.042) | (0.162) | (0.042) |
| revenue | -0.094 | -0.095 | -0.127 | -0.090 | -0.416* | -0.069 | -0.092 | -0.124 | -0.098 | -0.420* | -0.073 |
|  | (0.077) | (0.076) | (0.153) | (0.075) | (0.246) | (0.072) | (0.076) | (0.153) | (0.075) | (0.248) | (0.072) |
| market | -0.161* | -0.161* | -0.267 | -0.151 | 0.265 | -0.177* | -0.170* | -0.283 | -0.167* | 0.265 | -0.191** |
|  | (0.094) | (0.095) | (0.302) | (0.095) | (0.326) | (0.093) | (0.095) | (0.302) | (0.095) | (0.328) | (0.093) |
| Constant | -13.036*** | -13.297*** | 7.725* | -13.585*** | 3.209 | -13.496*** | -13.187*** | 8.291* | -13.270*** | 3.632 | -13.543*** |
|  | (1.895) | (1.847) | (4.405) | (1.833) | (4.713) | (1.806) | (1.831) | (4.368) | (1.816) | (4.721) | (1.794) |
| Observations | 1,110 | 1,110 | 1,110 | 1,110 | 1,110 | 1,110 | 1,110 | 1,110 | 1,110 | 1,110 | 1,110 |
| R-squared | 0.638 | 0.646 | 0.294 | 0.649 | 0.402 | 0.656 | 0.648 | 0.297 | 0.653 | 0.404 | 0.661 |
| F | 24.43 | 24.07 | 4.697 | 21.85 | 3.612 | 23.40 | 19.96 | 4.344 | 17.32 | 3.312 | 19.28 |
| Area FE | YES | YES | YES | YES | YES | YES | YES | YES | YES | YES | YES |
| Industry FE | YES | YES | YES | YES | YES | YES | YES | YES | YES | YES | YES |
| Year FE | YES | YES | YES | YES | YES | YES | YES | YES | YES | YES | YES |
