# Supplementary material for: RD internationalization, domestic technology alliance, and innovation in emerging market
Source: PLoS One. 2021 Jun 25;16(6):e0252669. doi: 10.1371/journal.pone.0252669 (PMC8232540; doi:10.1371/journal.pone.0252669)
Supplement: S7 Table — (DOCX) [file pone.0252669.s008.docx]

**S7 Table.** Robustness test results based on R&D internationalization variable substitution

|  | m1 | m2 | m3 | m4 | m5 | m6 | m7 | m8 | m9 | m10 | m11 |
| --- | --- | --- | --- | --- | --- | --- | --- | --- | --- | --- | --- |
| VARIABLES | patent | patent | doteal | patent | absorp | patent | patent | doteal | patent | absorp | patent |
| ovnrd |  | 0.114*** | 0.154*** | 0.098*** | 0.062*** | 0.110*** | 0.089*** | 0.156*** | 0.073*** | 0.063*** | 0.085*** |
|  |  | (0.013) | (0.018) | (0.014) | (0.014) | (0.013) | (0.014) | (0.019) | (0.014) | (0.017) | (0.014) |
| doteal |  |  |  | 0.040*** |  |  |  |  | 0.040*** |  |  |
|  |  |  |  | (0.015) |  |  |  |  | (0.015) |  |  |
| absorp |  |  |  |  |  | 0.055*** |  |  |  |  | 0.052*** |
|  |  |  |  |  |  | (0.014) |  |  |  |  | (0.014) |
| comp |  |  |  |  |  |  | 0.566** | 0.299 | 0.593** | 0.650* | 0.517** |
|  |  |  |  |  |  |  | (0.255) | (0.379) | (0.248) | (0.355) | (0.258) |
| c.c_ovnrd#c.comp |  |  |  |  |  |  | -0.078*** | 0.011 | -0.093*** | 0.002 | -0.079*** |
|  |  |  |  |  |  |  | (0.021) | (0.018) | (0.022) | (0.020) | (0.020) |
| doteal*comp |  |  |  |  |  |  |  |  | 0.047*** |  |  |
|  |  |  |  |  |  |  |  |  | (0.013) |  |  |
| absorp*comp |  |  |  |  |  |  |  |  |  |  | -0.018*** |
|  |  |  |  |  |  |  |  |  |  |  | (0.007) |
| size | 0.675*** | 0.662*** | -0.109*** | 0.666*** | -0.061** | 0.679*** | 0.676*** | -0.113*** | 0.673*** | -0.065** | 0.698*** |
|  | (0.044) | (0.036) | (0.039) | (0.036) | (0.029) | (0.036) | (0.036) | (0.039) | (0.035) | (0.029) | (0.036) |
| age | 0.017** | 0.017** | -0.011 | 0.018** | 0.007 | 0.014** | 0.014* | -0.012 | 0.015** | 0.007 | 0.013* |
|  | (0.008) | (0.007) | (0.007) | (0.007) | (0.005) | (0.007) | (0.007) | (0.007) | (0.007) | (0.005) | (0.007) |
| exper | 0.033*** | 0.020*** | -0.023** | 0.022*** | -0.013* | 0.020*** | 0.013* | -0.023** | 0.016** | -0.013* | 0.013** |
|  | (0.009) | (0.006) | (0.011) | (0.007) | (0.008) | (0.007) | (0.007) | (0.011) | (0.007) | (0.008) | (0.007) |
| roe | 0.185*** | 0.141*** | 0.129*** | 0.130** | -0.018 | 0.152*** | 0.157*** | 0.130*** | 0.153*** | -0.018 | 0.173*** |
|  | (0.055) | (0.050) | (0.038) | (0.051) | (0.029) | (0.050) | (0.054) | (0.038) | (0.054) | (0.029) | (0.053) |
| tobinq | -0.032 | -0.028 | 0.003 | -0.030 | 0.049*** | -0.049* | -0.028 | 0.003 | -0.029 | 0.049*** | -0.044 |
|  | (0.033) | (0.029) | (0.015) | (0.030) | (0.009) | (0.027) | (0.030) | (0.015) | (0.030) | (0.009) | (0.028) |
| cash | -0.028 | -0.038 | 0.011 | -0.034 | -0.002 | -0.040 | -0.035 | 0.011 | -0.030 | -0.003 | -0.035 |
|  | (0.035) | (0.030) | (0.029) | (0.028) | (0.027) | (0.031) | (0.029) | (0.029) | (0.028) | (0.026) | (0.030) |
| revenue | -0.133** | -0.110** | -0.034 | -0.107** | -0.098** | -0.080 | -0.107** | -0.033 | -0.108** | -0.099** | -0.086* |
|  | (0.053) | (0.052) | (0.046) | (0.052) | (0.040) | (0.052) | (0.052) | (0.046) | (0.052) | (0.040) | (0.052) |
| market | -0.117 | -0.023 | -0.118 | -0.009 | 0.082 | -0.041 | -0.031 | -0.125 | -0.013 | 0.081 | -0.045 |
|  | (0.110) | (0.102) | (0.111) | (0.100) | (0.061) | (0.101) | (0.100) | (0.112) | (0.097) | (0.061) | (0.098) |
| Constant | -8.780*** | -9.220*** | 4.243*** | -9.597*** | 1.833** | -9.527*** | -9.256*** | 4.590*** | -9.451*** | 2.111** | -9.848*** |
|  | (1.133) | (1.073) | (1.301) | (1.069) | (0.794) | (1.076) | (1.047) | (1.316) | (1.029) | (0.820) | (1.063) |
| Observations | 1,110 | 1,110 | 1,104 | 1,110 | 1,110 | 1,110 | 1,110 | 1,104 | 1,110 | 1,110 | 1,110 |
| Pseudo R2 | 0.7939 | 0.8125 | 0.1674 | 0.8146 | 0.1976 | 0.8168 | 0.8176 | 0.1678 | 0.8210 | 0.1994 | 0.8225 |
| Wald chi2 | 979.9 | 1296 | 97.70 | 1327 | 99.41 | 1329 | 1304 | 98.12 | 1359 | 104.3 | 1342 |
| Area FE | YES | YES | YES | YES | YES | YES | YES | YES | YES | YES | YES |
| Industry FE | YES | YES | YES | YES | YES | YES | YES | YES | YES | YES | YES |
| Year FE | YES | YES | YES | YES | YES | YES | YES | YES | YES | YES | YES |
